# High-Affinity NIR-Fluorescent Inhibitors for Tumor Imaging via Carbonic Anhydrase IX

**DOI:** 10.1021/acs.bioconjchem.4c00144

**Published:** 2024-05-15

**Authors:** Gediminas Žvinys, Agne Petrosiute, Audrius Zakšauskas, Asta Zubrienė, Alvilė Ščerbavičienė, Zane Kalnina, Edita Čapkauskaitė, Vaida Juozapaitienė, Aurelija Mickevičiu̅tė, Kirill Shubin, Švitrigailė Grincevičienė, Steponas Raišys, Kaspars Tars, Jurgita Matulienė, Daumantas Matulis

**Affiliations:** † Department of Biothermodynamics and Drug Design, Institute of Biotechnology, Life Sciences Center, Vilnius University, Saulėtekio 7, Vilnius LT-10257, Lithuania; ‡ Department of Biological Models, Institute of Biochemistry, Life Sciences Center, Vilnius University, Saulėtekio 7, Vilnius LT-10257, Lithuania; § Latvian Biomedical Research and Study Centre, Ratsupites 1 k-1, Riga LV-1067, Latvia; ∥ Latvian Institute of Organic Synthesis, Aizkraukles 21, Riga LV-1006, Latvia; ⊥ Institute of Photonics and Nanotechnology, National Center for Physical Sciences and Technology, Vilnius University, Saulėtekio 3, Vilnius LT-10257, Lithuania

## Abstract

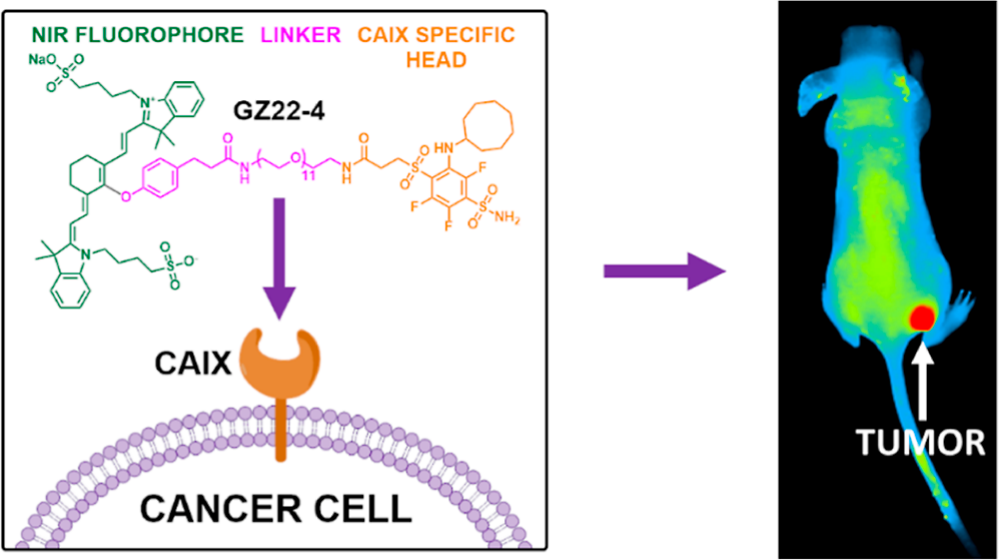

Tumor imaging and delivery of therapeutic agents may be achieved by designing high-affinity and high-selectivity compounds recognizing a tumor cell-expressing biomarker, such as carbonic anhydrase IX (CA IX). The CAIX, overexpressed in many hypoxic solid tumors, helps adjust to the energy requirements of the hypoxic environment, reduces intracellular acidification, and participates in the metastatic invasion of adjacent tissues. Here, we designed a series of sulfonamide compounds bearing CAIX-recognizing, high-affinity, and high-selectivity groups conjugated via a PEG linker to near-infrared (NIR) fluorescent probes used in the clinic for optically guided cancer surgery. We determined compound affinities for CAIX and other 11 catalytically active CA isozymes by the thermal shift assay and showed that the affinity *K*_d_ value of CAIX was in the subnanomolar range, hundred to thousand-fold higher than those of other CA isozymes. Similar affinities were also observed for CAIX expressed on the cancer cell surface in live HeLa cell cultures, as determined by the competition assay. The NIR-fluorescent compounds showed excellent properties in visualizing CAIX-positive tumors but not CAIX-negative knockout tumors in a nude mice xenograft model. These compounds would therefore be helpful in optically guided cancer surgery and could potentially be developed for anticancer treatment by radiotherapy.

## 1 Introduction

The development of theranostic fluorescence imaging agents and radiopharmaceuticals is an important trend in cancer diagnosis and treatment,^1^ and selecting a specific cancer-related protein target is essential for success. Tumor hypoxia is a predictor of worse outcomes and treatment resistance in a variety of solid tumors. Characterization and detection of hypoxic regions within solid tumor masses are important tasks. Therefore, a molecular imaging application, targeting proteins that serve as markers for tumor hypoxia is needed. Such imaging could help decide which patients will benefit from hypoxia-targeting therapy and help detect and follow the treatment response of the disseminated metastatic disease.^2^

An increased expression of carbonic anhydrase IX (CAIX) has been noted in a variety of cancers,^3^ and the development of CAIX recognition-based theranostic radiopharmaceuticals has become an attractive research venue.^1^ Numerous CAIX-targeted radionuclide therapy agents are currently undergoing various phases of clinical trials.^4^ This interest stems from their unique characteristics, in which chemically identical entities can be used for diagnosis and cancer treatment by incorporating various radionuclides. The most suitable design approach, proven in the field of CAIX-targeting theranostic molecules, involves the formation of a conjugate between an effective CAIX inhibitor and a metal chelator, tethered with a suitable linker.^5^ The ideal radiotracer must have high sensitivity and high specificity for CAIX-positive metastases. Small molecules are promising for therapeutic radiopharmaceuticals (^177^Lu, ^161^Tb, ^225^Ac, ^212^Pb, and others) since myelotoxicity is observed in most patients treated with a radioimmunoconjugate.^6^ While most studies with small-molecule conjugates are preclinical, several tracers have been tested in clinical trials^7,8^ with notable off-target uptake of these compounds related to nonspecific CA binding of acetazolamide. Therefore, more specific and more sensitive CAIX-recognizing radiotracer molecules are needed.

The first step in developing such theranostic agents should involve the design and synthesis of a highly specific CAIX-recognizing compound. Our laboratory has designed a number of such candidate compounds.^9−11^ We have selected the most promising compounds and tagged them to the NIR-fluorescent probes as the first step in assessing the specificity and biodistribution of these candidate probes for further development. Next, we assessed the targeting abilities of the new CAIX-specific NIR molecules in vitro and in vivo. We showed here that CAIX-recognizing compounds conjugated with NIR-fluorescent probes resulted in suitable probes for CAIX-positive tumor visualization in mice xenograft models.

## 2 Results

### 2.1 Design of High-Affinity Selective NIR-Fluorescent Inhibitors of CAIX

**Scheme 1:**
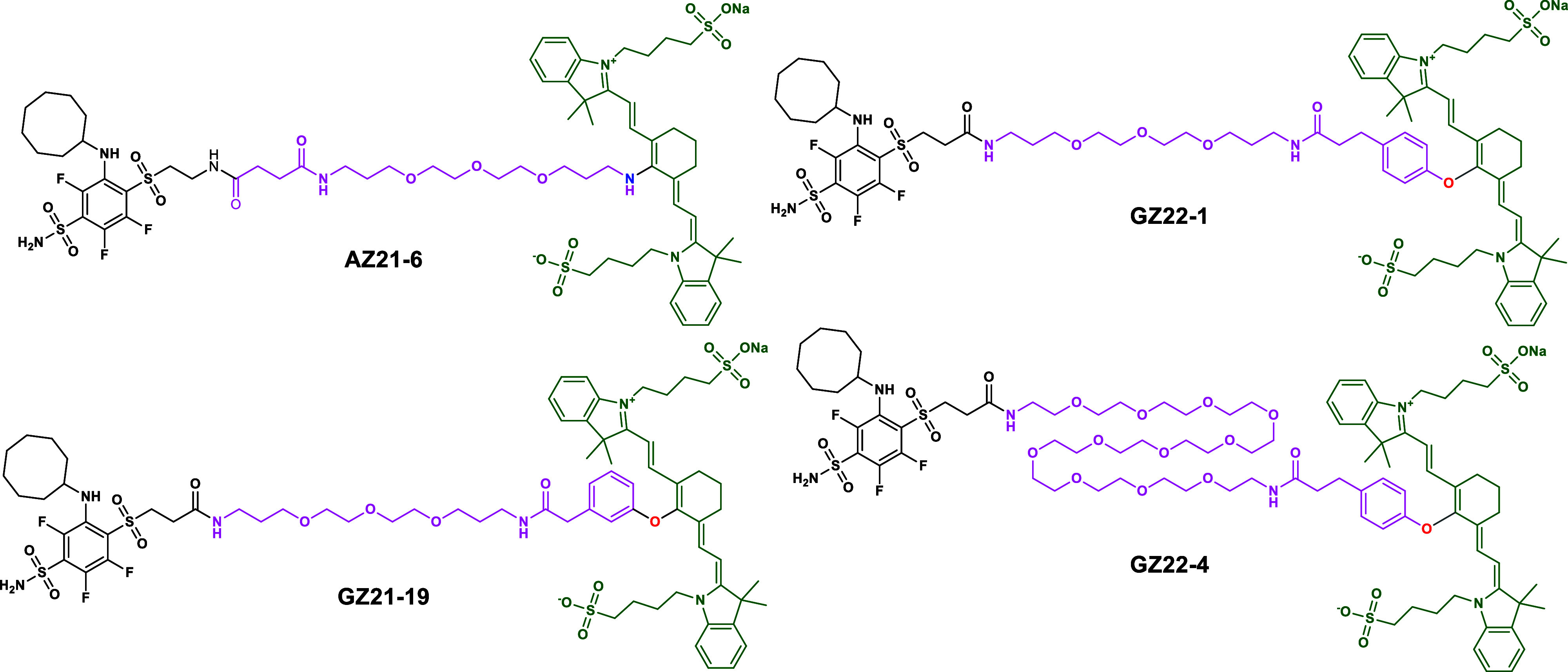
Chemical Structures of Compounds Bearing a CAIX-Recognizing Headgroup (on the Left of the Structure), a PEG-Containing Linker (Pink), and an NIR-Fluorescent Group (Green, on the Right of the Compound)

In search of ligands with high selectivity and affinity toward cancer-associated CAIX, our laboratory previously designed, synthesized, and tested over 1000 sulfonamide compounds. One of them, VD11-4-2, demonstrated a very strong binding affinity to CAIX (dissociation constant *K*_d_ = 30 pM) and remarkable selectivity over other CA isozymes.^9^ To make our inhibitor suitable for cancer imaging, we synthesized a compound **AZ21-6** that bears an NIR-783 fluorophore attached to the inhibitor via a 3-mer polyethylene glycol linker chain (marked in pink) via an amine group (marked in blue) (Scheme 1). Three additional compounds **GZ21-19**, **GZ22-1**, and **GZ22-4** were synthesized with oxygen instead of nitrogen connecting the NIR-783 fluorophore with the linker. We expected oxygen to have an impact on compounds’ NIR-fluorescence properties^12,13^ and not undergo a pH-related hypsochromic shift as reported with nitrogen compounds.^14^ Spectrophotometric data confirmed the desired bathochromic shift with all three compounds (Figure S1), and a stronger fluorescence response was also observed (Table S1). Compound **GZ22-4** was synthesized with a longer PEG linker of 11-mer than other compounds. A longer linker may be needed for a stronger fluorescence signal because of CAIX’s dimeric nature and potential shielding of the fluorophore connected with a short linker to the inhibitor while bound to an active site.^15^ A longer linker could provide the fluorophore with more flexibility and the ability to distance itself further from the protein. As expected, the compound **GZ22-4** was more brightly fluorescent than the other three compounds (Figure S4).

**Scheme 2:**
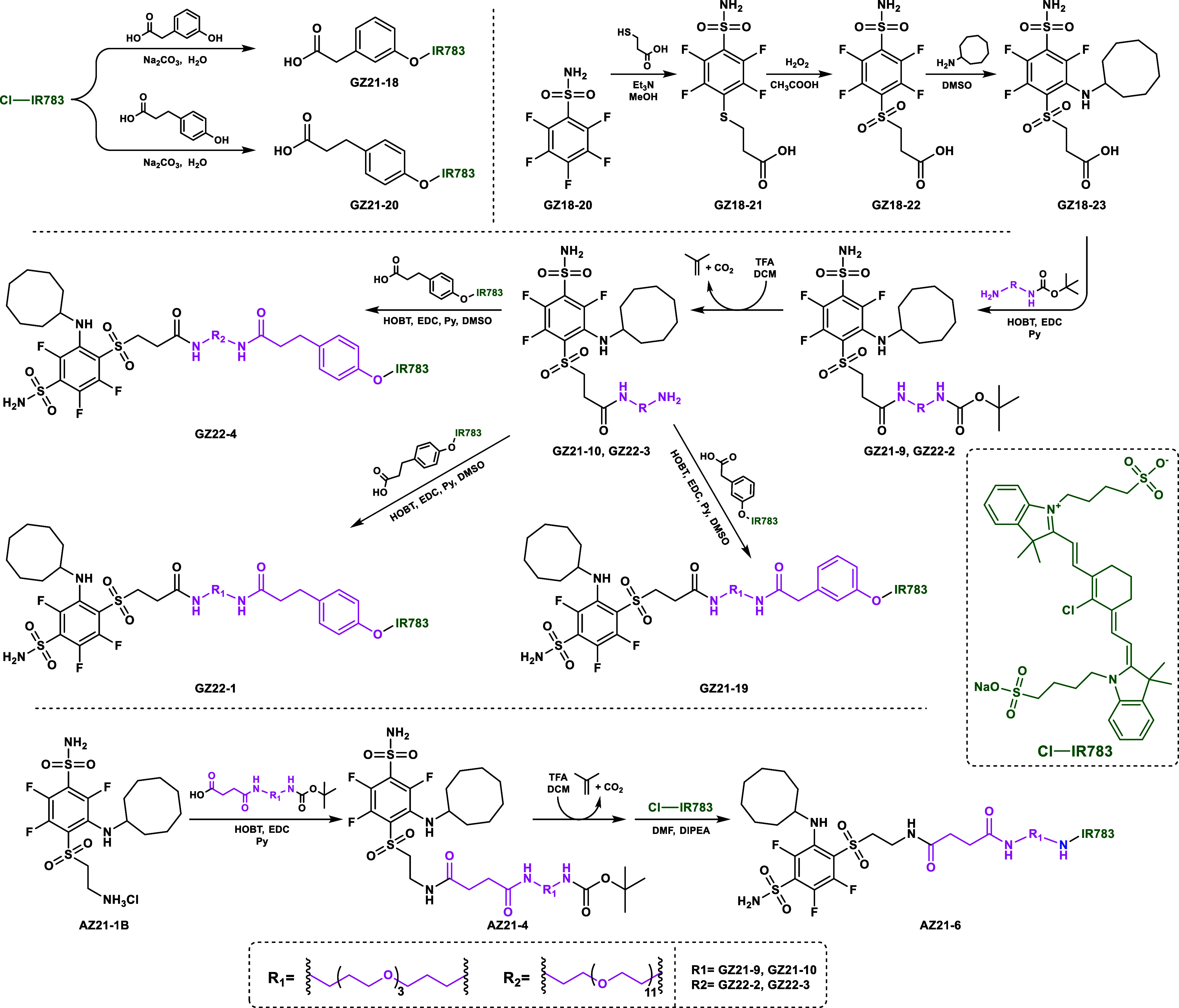
Scheme of the Synthesis of NIR-Fluorescent Compound

The synthesis of NIR-fluorescent compounds was carried out as shown in Scheme 2. The inhibitor **GZ18-23** and compounds leading to it were prepared by previously described procedures.^16^ The 3-mer and 11-mer PEG linkers were introduced via amide coupling. TFA and DCM were used for amine deprotection, yielding compounds **GZ21-10** and **GZ22-3**. The commercial NIR783 dye was coupled with phenolic alkyl acids. Final GZ compounds were acquired by amide coupling between phenolic NIR783 conjugates and PEGylated inhibitors. **AZ21-1B** was coupled with a 3-mer PEG linker via amide bond formation. Similarly, TFA and DCM were used for amine-group deprotection and subsequently coupled with NIR783, yielding the final **AZ21-6** compound.

### 2.2 Binding of NIR Inhibitors to CAIX and Other Recombinant Human CA Isozymes

**Figure 1:**
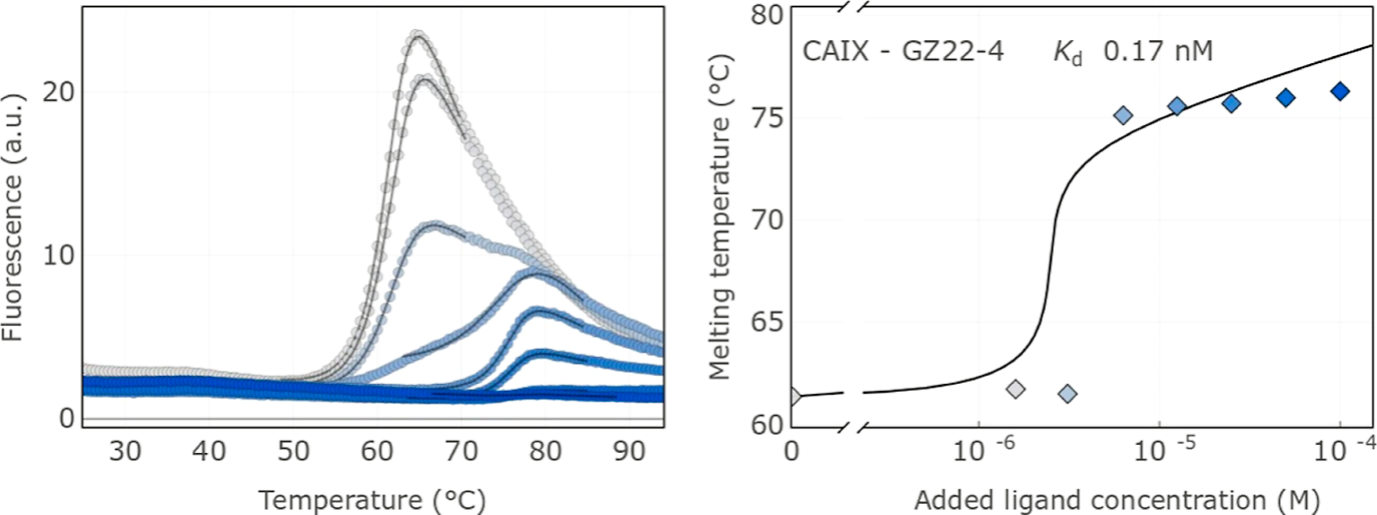
Determination of NIR-fluorescent compound binding affinities by the thermal shift assay [panels (A) and (B)]. (A) Fluorescence melting curves of CAIX at various concentrations of the added compound **GZ22-4**. (B) Dosing curve showing the *T*_m_ dependence on the compound concentration. The fit is performed automatically yielding the *K*_d_. Note that the data points near the inflection are not important for the curve fit to obtain a reliable *K*_d_.^17^

The binding affinities of the four NIR-fluorescent compounds to 12 catalytically active recombinant CA isozymes were determined by the fluorescence-based thermal shift assay (FTSA), and the dissociation constants (*K*_d_’s) at physiological pH (pH 7.0) and 37 °C are listed in Table 1. The curves of **GZ22-4** binding to CAIX are shown in Figure 1.

**Table 1:** Dissociation Constants *K*_d_’s (in nM Units) for the Interaction of Compounds with Human Recombinant CA Isozymes as Determined by FTSA at 37 °C and pH 7.0^a^

compound	*K*_d_, nM											
	CAI	CAII	CAIII	CAIV	CAVA	CAVB	CAVI	CAVII	**CAIX**	CAXII	CAXIII	CAXIV
**AZ21-6**	20,000	2000	30,000	8000	10,000	50	1000	60	**0.3**	50	10	100
**GZ21-19**	10,000	400	20,000	2000	2000	ND	700	ND	**0.2**	60	ND	100
**GZ22-1**	ND	20	30,000	7000	1000	ND	700	30	**0.3**	200	8	100
**GZ22-4**	ND	200	5000	8000	2000	ND	500	40	**0.2**	80	5	90
**GZ18-23**	5000	200	≥200,000	100	10,000	200	100	40	**0.03**	1.4	10	3

a ND—not determined. The *K*_d_ values for **GZ18-23** were taken from ref (16).

The thermal shift assay results show that all four NIR-fluorescent compounds bound with subnanomolar affinity to CAIX. A bulky PEG tail containing a NIR-fluorescent moiety only slightly diminished the affinity, as compared to a compound that does not possess the bulky group. In addition, all four compounds possessed high selectivity for CAIX over all the 11 remaining catalytically active human CA isozymes. Selectivity for binding CAIX over CAI, CAIII, CAIV, and CAVA exceeded 10,000-fold, while the selectivity over CAII, CAVB, CAVI, CAVII, CAXII, and CAXIV exceeded 100-fold and that over CAXIII exceeded 20-fold. Therefore, all four NIR-fluorescent compounds were high-affinity and strong-selectivity ligands suitable for further application for visualization of CAIX in cell cultures and live animals.

### 2.3 NIR-Fluorescent Inhibitor Binding to Live HeLa Cells Expressing CAIX

To measure NIR-fluorescent compound binding to CAIX expressed in cancer cells, the **GZ22-4** compound was applied in different concentrations to live HeLa cells grown under hypoxia and normoxia, as described in the Materials and Methods section, and was previously performed for the fluorescein-conjugated CAIX inhibitor **GZ19-32**.^16^ In contrast to **GZ19-32**, **GZ22-4** compound showed no difference in the fluorescence signal in hypoxia vs normoxia, most likely due to the lower fluorescence quantum yield of NIR dye as compared to fluorescein. The difference was hidden in the low signal-to-noise ratio.

**Figure 2:**
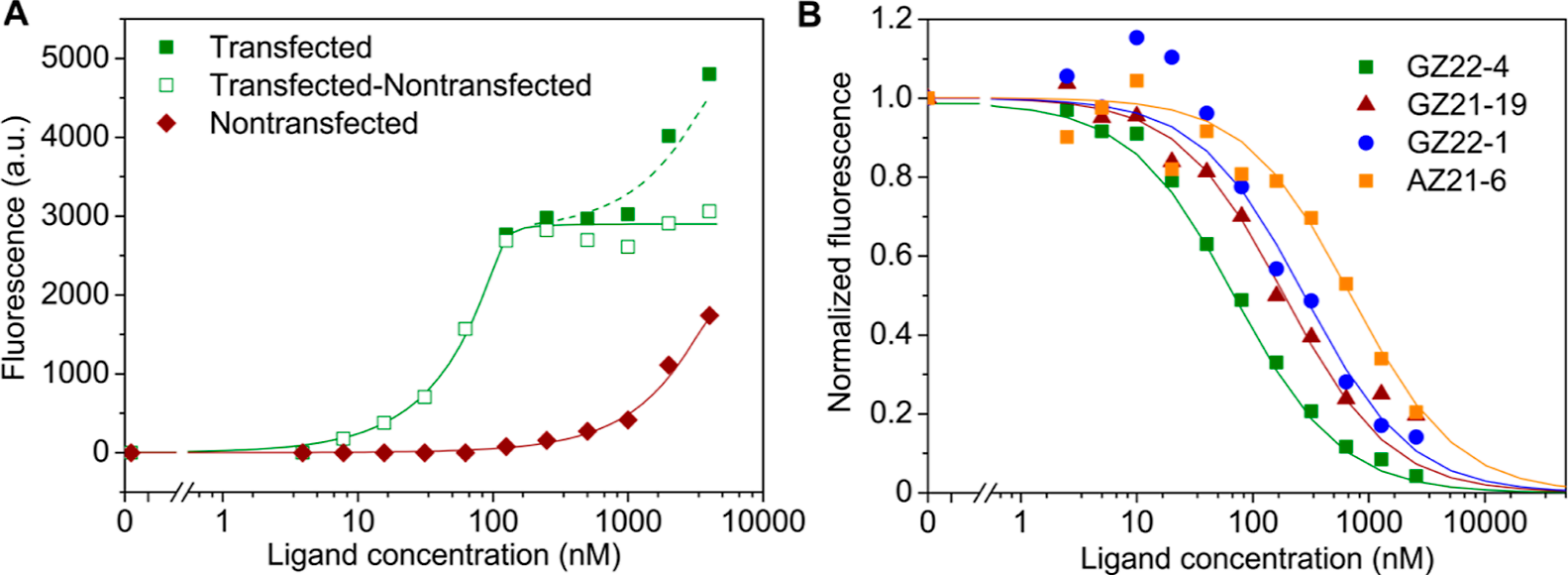
(A) Dosing curves with the NIR-fluorescent **GZ22-4** compound of HeLa cell culture grown for 2 days under normoxia, nontransfected (brown filled diamonds) or transfected with the CAIX-encoding plasmid (green filled squares). Nonspecific binding of the dye is visible at high concentrations exceeding approximately 1 μM for both transfected and nontransfected cell cultures. Specific CAIX binding is seen only for transfected cells. Subtraction of nontransfected from transfected fluorescence values yielded open data points, fit to the regular dosing model (solid green line). The fit shows that there is 120 nM CAIX concentration in the cell culture. (B) Determination of affinities of compounds **GZ22-4** (green squares), **GZ21-19** (red triangles), **GZ22-1** (blue circles), and **AZ21-6** (orange squares) for cell-expressed CAIX via a competition assay with the fluorescein-labeled **GZ19-32** compound. The cells were grown under hypoxia and not transfected because fluorescence properties of **GZ19-32** were sufficient to determine CAIX expression in hypoxic cells.

To increase the amount of CAIX expression, the HeLa cells were transfected with the CAIX-encoding plasmid pCMV-CAIX. There was a dramatic increase in CAIX expression observed both in the dosing curve and fluorescence microscopy results. Figure 2A shows the dosing curves of **GZ22-4** obtained with the transfected and nontransfected HeLa cell cultures. In the case of transfection, we can see both the specific CAIX-dependent signal and relatively weak nonspecific binding of the dye to unknown targets at concentrations above approximately 1 μM (shown as a dashed curve, increasing at the same concentrations as the nontransfected cell dosing curve).

Fluorescence values corresponding to the transfection effect were obtained by subtraction of nontransfected values from transfected fluorescence values, showing the resultant data points as open squares. These data were fit to a regular dosing curve yielding the CAIX concentration 120 nM and *K*_d_ equal to 1 nM. These data match well with the *K*_d_ obtained by FTSA for purified CAIX, equal to 0.2 nM. However, the determination of the *K*_d_ via dosing curves at such high protein concentrations is not accurate and can only serve as an estimate of affinity.

### 2.4 Affinities of NIR-Fluorescent Compounds for Cells Expressing CAIX by the Competition Assay

Hypoxia-grown HeLa cell culture expressed approximately 2 nM CAIX, which was quantified by fluorescein-labeled **GZ19-32** added at a constant concentration of 10 nM throughout the assay. The four NIR-fluorescent compounds were dosed and competed with the **GZ19-32** compound in a dose-dependent manner. At high concentrations, all four compounds fully outcompeted the fluorescein-bearing compound yielding nice competition curves (Figure 2B). Each curve was fit using the total protein CAIX (*P*_t_) value of 2 nM, and the *K*_d_ of **GZ19-32** was 200 pM. The dissociation constants for each NIR-fluorescent compound were 4 nM for **GZ21-19**, 6 nM for **GZ22-1**, 1.5 nM for **GZ22-4**, and 15 nM for **AZ21-6**. Thus, compound affinities were similar but not identical, despite having identical headgroups. The differences in the tail length and chemical structure must have caused the small differences in affinities, where **GZ22-4** showed the highest affinity and **AZ21-6** the lowest affinity of the four. The affinity of fluorescein-bearing **GZ19-32** was the highest of all, around 200 pM.

Note that there was no need for transfection for the competition assay because the levels of hypoxia-induced CAIX were sufficient to be quantified by highly fluorescent **GZ19-32**. However, the NIR compounds could not be used to quantify CAIX expressed in cells grown under hypoxia in the plate reader because fluorescence detection was too low in the NIR region. The NIR-fluorescent compounds did not interfere with readings in the green fluorescence range since they fluoresce in the NIR region around 850 nm, while **AZ21-6** has fluorescence maximum around 780 nm.

### 2.5 Microscopy of the NIR-Fluorescent Compound Staining of CAIX-Expressing Human Cancer Cells

**Figure 3:**
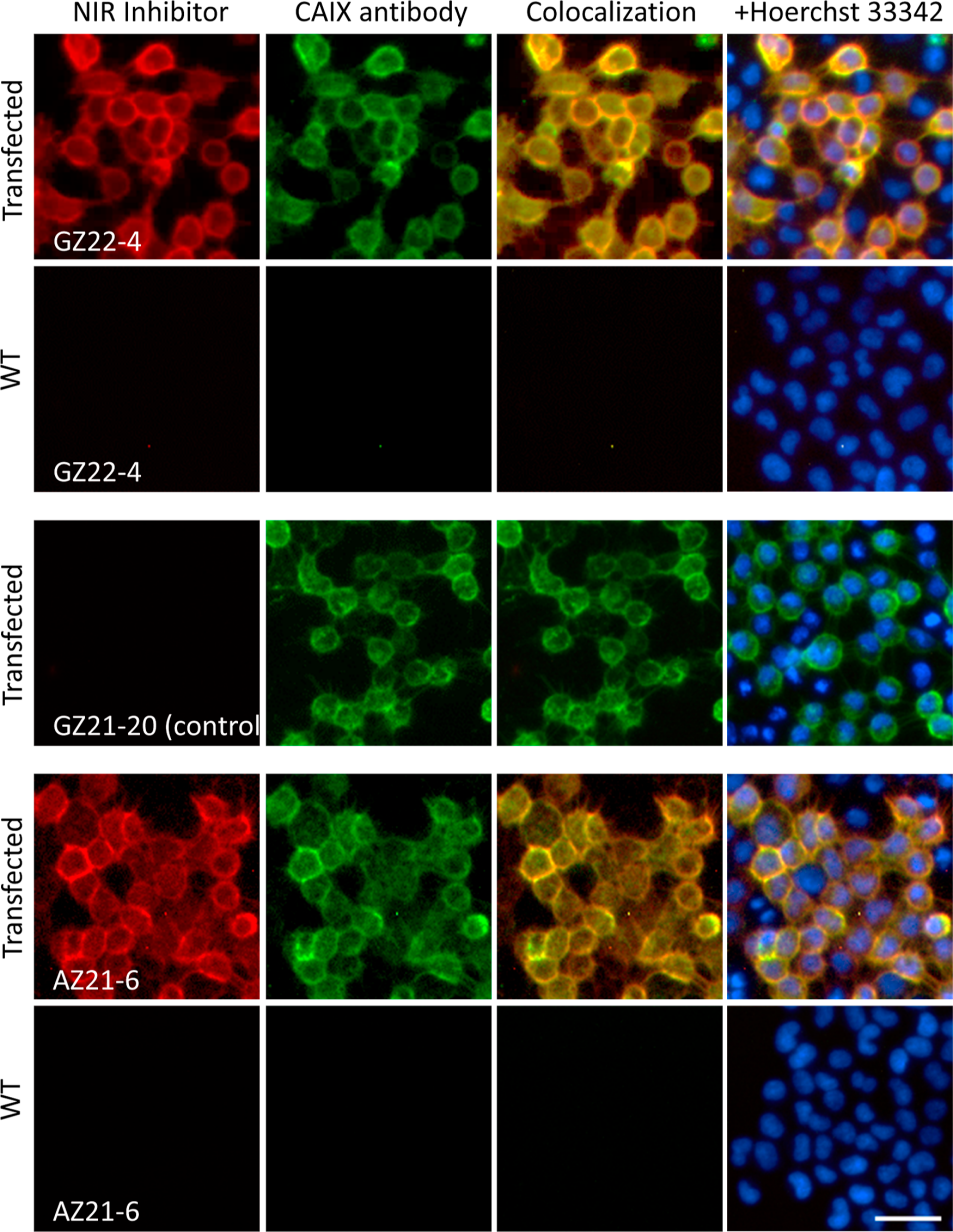
Staining of live HeLa cells grown under normoxia (wild type and transfected with CAIX-encoding DNA) and incubated with 250 nM NIR inhibitors (**GZ22-4**, **AZ21-6**) and control compound **GZ21-20** that does not contain the CAIX-recognizing headgroup. Beginning from the left, the columns show the binding of NIR inhibitors (red), CAIX antibody (green), colocalization of NIR compounds and CAIX antibody (first two columns), and colocalization overlaid with cell nuclei (Hoerchst 33342, blue). Due to approximately 80% transfection efficiency, some cells remained untransfected and showed no antibody or compound binding. Scale bar length is 40 μm.

Live HeLa cell culture was used for visualization of the NIR-fluorescent compound binding to CAIX expressed on cell membranes (Figures 3 and S2). All four CAIX-selective compounds (**GZ22-4**, **AZ21-6**, **GZ21-19**, and **GZ22-1**) stained the membranes of pCMV-CAIX-transfected cells, while wild-type cells, grown both under normoxia and hypoxia, gave no visual signal (1st column, Figures 3 and S2). Hypoxic conditions were insufficient for CAIX-specific fluorescence detection (Figure S3). Transfected cells expressed 10-fold more CAIX protein than cells grown under hypoxia, as determined by dosing the fluorescein-containing compound **GZ19-32**. The control compound **GZ21-20** that does not have CAIX-specific inhibitor headgroup shows no visual response on transfected cells (Figure 3). The second column shows cell staining with the CAIX antibody, which colocalized with the staining of NIR-fluorescent compounds (3rd column). The fourth column shows the overlay of colocalization with the staining of cell nuclei. The **GZ22-4** showed the best fluorescence signal as compared to the other three compounds if images were taken at identical exposure conditions (Figure S4). We should point out that microphotographs in Figures 3 and S2 were taken at different exposure times to ensure good visualization quality. Nevertheless, all four compounds were suitable for direct visualization of CAIX expressed on transfected cell membranes.

### 2.6 CAIX-Expressing Tumor Imaging in HeLa Xenograft-Based Mice

Recognition of CAIX-expressing tumors by a specific NIR probe in vivo requires a suitable xenograft platform. Previously, our laboratory has developed a CAIX-knockout HeLa cell line (HeLa CAIX^KO^), confirmed by sequencing and several antibody-based detection methods^16^.

**Figure 4:**
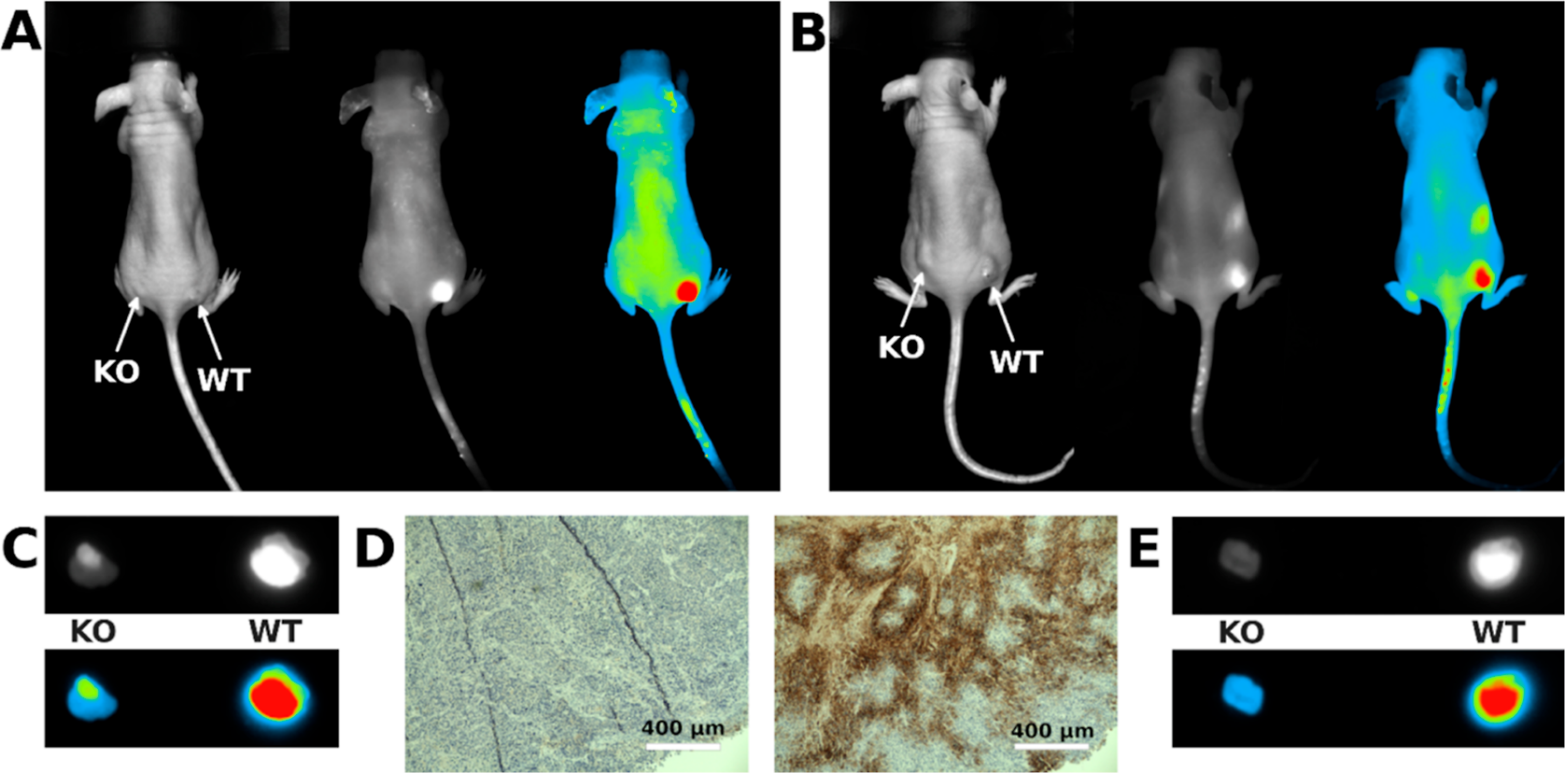
**GZ22-4** and **AZ21-6** distribution patterns in nude mice with Hela xenograft. (A) **GZ22-4** compound uptake in nude mice bearing HeLa CAIX^WT^ tumors injected into the right flank compared to HeLaCAIX^KO^ tumors, injected into the left flank. (B) **AZ21-6** compound uptake in nude mice bearing HeLa CAIX^WT^ tumors injected into the right flank compared to HeLaCAIX^KO^ tumors, injected into the left flank. (C) **GZ22-4** compound uptake differences in HeLa CAIX^WT^ compared to HeLaCAIX^KO^ tumors ex vivo. Images were obtained 3 days after dye injection. (D) Immunohistochemistry of HeLaCAIX^WT^ versus HeLaCAIX^KO^ line xenograft tumor samples reveal the absence of CAIX staining in HeLa CAIX^KO^ tumors compared to HeLaCAIX^WT^. Scale bars, 400 μm. (E) **AZ21-6** compound uptake differences in HeLa CAIX^WT^ compared to HeLaCAIX^KO^ tumors ex vivo. Images were obtained 7 days after dye injection.

The HeLaCAIX^WT^ and HeLaCAIX^KO^ cells were injected subcutaneously (S.Q.) into opposite flanks of nude mice, 3 × 10^6^ cells per injection. Once tumors reached the palpable size, we performed imaging using the UVITEC AllianceQ9 Imager system. The images were obtained every 24 h after intravenous (I.V.) compound injection into a tail vein. To analyze fluorescent compound uptake, two best candidate compounds were chosen, **AZ21-6** and **GZ22-4**. Compound **GZ22-4** was selected because of the photostability and good quantum yield (Table S1), and the fluorescence maximum of **AZ21-6** closely resembled the center of the NIR filter of the imaging system. Both compounds had the best solubility properties and good in vitro binding to cells. We saw a preferential uptake of the compound in HeLaCAIX^WT^ tumors compared to HeLaCAIX^KO^ tumors when using both compounds (Figure 4A,B). The **GZ22-4** NIR compound seemed to be slightly more specific toward CAIX, consistent with the highest affinity for CAIX (Figure 2) and had a faster clearance from nontumor locations based on daily images.

## 3 Discussion

The NIR-fluorescent compounds described in this study bound to CAIX with the highest affinity for CAIX among all small-molecule compounds described in the literature. Taking into account their high selectivity for CAIX over remaining CA isozymes makes them highly preferable for CAIX tumor visualization. Chemically, the compounds are rather simple to synthesize, and they have good physicochemical properties such as aqueous solubility.

Affinity together with selectivity is the main reason why these compounds in the in vivo study showed so significantly different uptake of CAIX-NIR compounds in HeLaCAIX^WT^ tumors as compared to HeLaCAIX^KO^ tumors. It is important to emphasize that both tumors were in the same mouse, making the effect independent of individual mouse variation. The NIR-fluorescent compounds were specific toward CAIX and at the shown times labeled specifically the WT tumor bearing CAIX. Furthermore, based on the mice daily images, they had a relatively fast clearance from nontumor organs. Still, there was somewhat prolonged accumulation of both tested compounds in the liver and kidney. Despite quite high selectivity, the headgroup recognizing CAIX that bears the cyclo-octyl ring was not completely selective for CAIX. Several other CA isozymes bound the compounds with nanomolar affinity, among which are CAII, CAVB, CAVII, CAXII, CAXIII, and CAXIV. Some of those interactions, especially with CAXII, could be beneficial, but others may lead to nonspecific binding to other CA isozymes and staining nontumor tissues if applied at concentrations exceeding the CAIX amount in the tumor. Thus, a further increase in selectivity could be beneficial for such compound application in theranostics of CAIX-positive cancers.

Since the discovery of CAIX as a hypoxic tumor biomarker,^18−20^ numerous studies have used antibodies, small-molecule compounds, and nanoparticles conjugated to fluorescent or radioactive elements designed both for tumor imaging and cancer treatment.^1−3,5,14,21−54^ Several small-molecule probes have reached clinical trials. For instance, ^18^F-VM4-037, a small-molecule radiotracer developed on the sulfonamide pharmacophore, a derivative of the CA ligand ethoxzolamide, failed to localize the ccRCC tumor as it had a high background in the kidney and liver.^29^ This was partially due to ethoxzolamide-limited selectivity for CAIX over other CA isozymes. The study with a ^99m^Tc-labeled acetazolamide derivative, ^99m^Tc-PHC-102, showed encouraging results in a pilot study using a SPECT/CT scanner for identifying substantial tumor and metastasis uptake. However, some gallbladder and stomach uptake was noted.^7^ A pilot study of ^68^Ga-NY104, based on an acetazolamide core and connected to a hydrophilic spacer and a chelator NOTA in 3 patients with ccRCC showed excellent tumor uptake and a high ratio of tumor-to-background. Non-negligible uptake in the kidney, stomach, intestine, lung, and liver was also noted.^8^ Most of the conjugated compounds were based on an acetazolamide or a benzenesulfonamide headgroup that possesses limited selectivity for CAIX. The main issue appeared to be insufficient affinity and the lack of selectivity in binding CAIX over other CA isozymes and potentially other proteins.

Contrary to our previously designed fluorescein-labeled compounds that nicely stained hypoxia-grown cell cultures and could be used to quantify the expression of the CAIX protein,^16^ the same cells stained with the NIR fluorescent compounds did not show any detectable signal. This was due to limited capabilities of detection in the infrared wave region. We estimated that the fluorescence yield was 10-fold lower for NIR compounds compared to fluorescein-bearing **GZ19-32**. In addition, the Cy7 filter of the fluorescence microscope used for the study was not fully suited for the detection in the 850 nm wavelength region. However, the NIR-compound-stained tumors in mice were nicely visible due to a multilayer cell structure of the mice tumors and better-suited NIR detection filters of the mice imaging system.

The NIR-fluorescent compounds exhibited extremely high affinity, where the *K*_d_ was 0.2 to 0.3 nM as determined by the fluorescence thermal shift assay. In addition, compound affinities were determined by our previously developed competition assay performed with live cells.^16^ The *K*_d_ values obtained by the competition assay showed somewhat lower affinities for cell-expressed CAIX than for purified CAIX. The difference was approximately 10-fold, which was likely caused by the presence of various factors and proteins in the growth medium and cells.

Such high affinity was possibly necessary for optimal imaging, but it delayed the clearance of the compound from nontumor areas, and thus it took several days for optimal visualization of the WT tumor. The balance between affinity and selectivity is crucial for successful imaging of the tumor. Sufficiently good imaging could also be obtained with probes exhibiting lower affinities for CAIX, as shown in numerous studies, thus explaining visualization results after applying nonselective benzenesulfonamide- or acetazolamide-based conjugated compounds for CAIX imaging. However, this could be shown only because CAIX is expressed on the cell surface, while other CA isozymes are inside cells, thus minimizing nonspecific interactions. In our opinion, compounds possessing higher affinity for CAIX and sufficient selectivity over remaining CA isozymes, such as **GZ22-4**, are advantageous to visualize CAIX-positive tumors and could be further developed by attaching PET probes, other radioactive elements, for both visualization and cancer treatment.

## 4 Materials and Methods

### 4.1 Chemical Synthesis

All starting materials and reagents were commercial products and were used without further purification. Melting points of the compounds were determined in open capillaries on a Thermo Scientific 9100 Series and were uncorrected. Column chromatography was performed using silica gel 60 (0.040–0.063 mm, Merck). ^1^H and ^13^C NMR spectra were recorded on a Bruker Ascend 400 spectrometer (400 and 100 MHz, respectively) with TMS as an internal standard, and proton and carbon chemical shifts are expressed in parts per million (ppm) in the indicated solvent. ^19^F NMR spectra were recorded on a Bruker Ascend 400 spectrometer (376 MHz) with CFCl_3_ as an internal standard, and fluorine chemical shifts are expressed in parts per million (ppm) in the indicated solvent. Multiplicity was defined as s (singlet), d (doublet), t (triplet), q (quartet), dd (double doublet), ddd (double double doublet), m (multiplet), and br s (broad singlet). TLC was performed with silica gel 60 F254 aluminum plates (Merck) and visualized with UV light. High-resolution mass spectroscopy (HRMS) spectra were recorded by an Agilent TOF 6230 equipped with an Agilent Infinity 1260 HPLC system, in positive or negative electrospray ionization (ESI) mode. In the positive mode, isocratic elution of 95% acetonitrile w/5% of 1% formic acid solution in water (18.2 MΩ·cm@25 °C) was used, and in the negative mode 80% acetonitrile w/20% water at a flow rate of 0.300–0.500 mL/min was used. Absorption was measured using a PerkinElmer Lambda 950 spectrophotometer and a 1 mm fused silica cuvette. Emission was measured using a Hamamatsu PMA-12 multichannel spectrometer and a 1 mm fused silica cuvette at 45° excitation angle. Excitation source was an NKT Photonics FIANIUM-15 laser coupled to a SuperK SELECT multichannel filter working at 78 MHz repetition rate. The photoluminescence quantum yield was estimated by utilizing an integrating sphere (SphereOptics) coupled to the same PMA-12 CCD spectrometer via an optical fiber, where a 150 W xenon lamp (LOT-Oriel) coupled to a monochromator (Sciencetech Inc.) was used as an excitation source.

The synthesis of compounds **GZ18-20**, **GZ18-21**, **GZ18-22**, and **AZ21-1B** has been previously described in the literature.^16^

### 4.2 3-((2-(Cyclooctylamino)-3,5,6-trifluoro-4-sulfamoylphenyl)sulfonyl)propanoic Acid (**GZ18-23**)^55^

Purified with column chromatography (EtOAc/CHCl_3_/AcOH, 49.5:49.5:1 v/v/v). Yield: 3.3 g, 54%; mp 143–144 °C; TLC (EtOAc/CHCl_3_/AcOH, 49.5:49.5:1 v/v/v): *R*_f_ = 0.58.

### 4.3 General Procedure for the Syntheses of **GZ21-9** and **GZ22-2**

A mixture of **GZ18-23** (200.0 mg, 0.42 mmol, 1 equiv for **GZ21-9**; 97.0 mg, 0.21 mmol, 1 equiv for **GZ22-2**), EDC (196.0 mg, 1.27 mmol, 3 equiv for **GZ21-9**; 90.0 mg, 0.58 mmol, 2.8 equiv for **GZ22-2**), HOBT (114.0 mg, 0.85 mmol, 2 equiv for **GZ21-9**; 58.6 mg, 0.43 mmol, 2.1 equiv for **GZ22-2**), *tert*-butyl(3-(2-(2-(3-aminopropoxy)ethoxy)ethoxy)propyl)carbamate (203.0 mg, 0.63 mmol, 1.5 equiv) for **GZ21-9** or *tert*-butyl(35-amino-3,6,9,12,15,18,21,24,27,30,33-undecaoxapentatriacontyl)carbamate (200.0 mg, 0.31 mmol, 1.5 equiv) for **GZ22-2**, and 5 mL of dry pyridine was stirred at room temperature under an argon atmosphere for 24 h. The reaction progress was monitored using TLC. Solvent was evaporated under reduced pressure, the crude product was poured into saturated sodium chloride solution (10 mL), acidified until neutral pH, and extracted with ethyl acetate. The organic layer was washed with sat. sodium chloride solution and dried over sodium sulfate. The solvent was evaporated under reduced pressure, and the residue was purified with column chromatography.

### 4.4 *tert*-Butyl (17-((2-(Cyclooctylamino)-3,5,6-trifluoro-4-sulfamoylphenyl)sulfonyl)-15-oxo-4,7,10-trioxa-14-azaheptadecyl)carbamate (**GZ21-9**)

TLC (EtOAc/AcOH, 99:1 v/v): *R*_f_ = 0.5. Purified with column chromatography (CHCl_3_/EtOAc, 1:1 v/v, then 100% EtOAc). Yield: 0.12 g, 37%; mp 70–71 °C;

^1^H NMR (400 MHz, DMSO-*d*_6_): δ 1.37 (9H, s, *tert*-Bu), 1.42–1.70 (16H, m, cyclooctane (12H) and PEG NHCH_2_C*H*_*2*_ (4H)), 1.80–1.89 (2H, m, cyclooctane), 2.57 (2H, t, *J* = 7.1 Hz, C*H*_*2*_CONH), 2.95 (2H, q, *J* = 6.7 Hz, PEG C*H*_*2*_NHCOO), 3.01 (2H, q, *J* = 6.7 Hz, PEG CH_2_CONHC*H*_*2*_), 3.33–3.41 (4H, m (inside H_2_O), NHCH_2_CH_2_C*H*_*2*_), 3.48 (8H, m, OC*H*_*2*_C*H*_*2*_O), 3.74 (2H, t, *J* = 7.1 Hz, SO_2_C*H*_*2*_), 3.75–3.81 (1H, m, C*H* cyclooctane), 6.60 (1H, d, *J* = 8.3 Hz, N*H* cyclooctylamine), 6.73 (1H, t, *J* = 5.4, N*H*COO), 8.03 (1H, t, *J* = 5.5, CH_2_CON*H*), 8.35 (2H, s, N*H*_*2*_SO_2_).

^13^C NMR (100 MHz, DMSO-*d*_6_): δ 23.27 (cyclooctane), 25.46 (cyclooctane), 27.21 (cyclooctane), 28.27 (*C*H_2_CON), 28.71 (*tert*-Bu), 29.54 (*C*H_2_CH_2_NHCOO), 30.17 (CONHCH_2_*C*H_2_), 32.67 (cyclooctane), 36.49 (*C*H_2_NHCOO), 37.68 (CONH*C*H_2_), 53.11 (SO_2_*C*H_2_), 55.88 (*C*H cyclooctane, d, *J*(^19^F–^13^C) = 11.0 Hz), 68.35 (*C*H_2_CH_2_CH_2_NHCOO), 68.55 (CONHCH_2_CH_2_*C*H_2_), 69.98 (O*C*H_2_CH_2_O), 70.21 (OCH_2_*C*H_2_O), 77.88 (*C*(CH_3_)_3_), 114.72–114.98 (m, *C1*), 127.80–128.19 (m, *C4*), 135.27 (d, *C2*, *J*(^19^F–^13^C) = 14.3 Hz), 137.21 (dm, *C5*, *J*(^19^F–^13^C) = 242.3 Hz), 144.45 (d, *C3*, *J*(^19^F–^13^C) = 250.6 Hz), 146.22 (C6, dm, *J*(^19^F–^13^C) = 251.6 Hz), 156.04 (*C*OO), 168.16 (*C*ON);

^19^F NMR (376 MHz, DMSO-*d*_6_): δ −125.03 (C3–*F*, s), −133.90 (C5–*F*, dd, ^1^*J* = 27.0 Hz, ^2^*J* = 12.4 Hz), −150.62 (C6–*F*, dd, ^1^*J* = 26.8 Hz, ^2^*J* = 6.6 Hz).

HRMS (*m*/*z*): [M]^+^ calcd for C_32_H_54_F_3_N_4_O_10_S_2_^+^, 775.3228; found, 775.3251.

### 4.5 *tert*-Butyl (39-((2-(Cyclooctylamino)-3,5,6-trifluoro-4-sulfamoylphenyl)sulfonyl)-37-oxo-3,6,9,12,15,18,21,24,27,30,33-undecaoxa-36-azanonatriacontyl)carbamate (**GZ22-2**)

TLC (acetone/EtOAc, 1:1 v/v): *R*_f_ = 0.15. Purified with column chromatography (acetone/EtOAc, 1:1 v/v → acetone → acetone/MeOH, 10:1 v/v). Yield: 0.14 g, 69%; mp 74–75 °C;

^1^H NMR (400 MHz, DMSO-*d*_6_): δ 1.37 (9H, s, *tert*-Bu), 1.42–1.69 (12H, m, cyclooctane), 1.79–1.89 (2H, m, cyclooctane), 2.60 (2H, t, *J* = 7.2 Hz, C*H*_*2*_CONH), 3.06 (2H, q, *J* = 6.0 Hz, PEG C*H*_*2*_NHCOO), 3.14 (2H, q, *J* = 5.8 Hz, PEG CH_2_CONHC*H*_*2*_), 3.47–3.52 (44H, m, PEG), 3.73 (2H, t, *J* = 7.2 Hz, SO_2_C*H*_*2*_), 3.75–3.82 (1H, m, C*H* cyclooctane), 6.60 (1H, d, *J* = 8.4 Hz, N*H* cyclooctylamine), 6.75 (1H, t, *J* = 5.8, N*H*COO), 8.15 (1H, t, *J* = 5.6, CH_2_CON*H*), 8.35 (2H, s, N*H*_*2*_SO_2_);

^13^C NMR (100 MHz, DMSO-*d*_6_): δ 23.28 (cyclooctane), 25.46 (cyclooctane), 27.21 (cyclooctane), 28.22 (*C*H_2_CON), 28.69 (*tert*-Bu), 32.69 (cyclooctane), 53.13 (SO_2_*C*H_2_), 55.88 (*C*H cyclooctane, d, *J*(^19^F–^13^C) = 11.1 Hz), 69.41 (PEG), 69.63 (PEG), 69.97 (PEG), 70.06 (PEG), 70.12–70.38 (m, PEG), 78.03 (*C*(CH_3_)_3_), 114.85 (dd, *C1*,^*1*^*J*(^19^F–^13^C) = 13.0 Hz, ^*2*^*J*(^19^F–^13^C) = 5.5 Hz), 128.01 (dd, *C4*, ^*1*^*J*(^19^F–^13^C) = 18.6 Hz, ^*2*^*J*(^19^F–^13^C) = 14.2 Hz), 135.29 (d, *C2*, *J*(^19^F–^13^C) = 12.9 Hz), 137.18 (dm, *C5*, *J*(^19^F–^13^C) = 246.3 Hz), 144.48 (d, *C3*, *J*(^19^F–^13^C) = 254.6 Hz), 146.18 (C6, dm, *J*(^19^F–^13^C) = 251.8 Hz), 156.04 (*C*OO), 168.39 (*C*ON);

^19^F NMR (376 MHz, DMSO-*d*_6_): δ −125.00 (C3–*F*, s), −133.97 (C5–*F*, dd, ^1^*J* = 26.9 Hz, ^2^*J* = 12.6 Hz), −150.60 (C6–*F*, dd, ^1^*J* = 27.0 Hz, ^2^*J* = 6.6 Hz).

HRMS (*m*/*z*): [M]^+^ calcd for C_46_H_82_F_3_N_4_O_18_S_2_^+^, 1099.5013; found, 1099.5025.

### 4.6 General Procedure for the Syntheses of **GZ21-10** and **GZ22-3**

**GZ21-9** (120.0 mg, 0.15 mmol, 1 equiv) or **GZ22-2** (130.0 mg, 0.12 mmol, 1 equiv) was dissolved in 1 mL of dichloromethane and added dropwise into trifluoracetic acid (4.5 g, 39.20 mmol, 260 equiv) precooled to 0 °C and then stirred at room temperature for 1 h. The reaction progress was monitored using TLC. The solvent was evaporated under reduced pressure, and the crude product was used for the next step without any additional purification.

### 4.7 *N*-(3-(2-(2-(3-Aminopropoxy)ethoxy)ethoxy)propyl)-3-((2-(cyclooctylamino)-3,5,6-trifluoro-4-sulfamoylphenyl)sulfonyl)propanamide (**GZ21-10**)

TLC (EtOAc): *R*_f_ = 0.1. Yield: 99.2 mg, 95%; yellow oily residue;

^1^H NMR (400 MHz, DMSO-*d*_6_): δ 1.47–1.75 (14H, m, cyclooctane (12H), PEG C*H*_*2*_CH_2_NH_3_), 1.80–1.85 (4H, m, cyclooctane (2H), PEG NHCH_2_C*H*_*2*_), 2.64 (2H, t, *J* = 7.1 Hz, C*H*_*2*_CONH), 2.89 (2H, s, *J* = 6.6 Hz, PEG C*H*_*2*_NH_3_), 3.08 (2H, q, *J* = 6.1 Hz, PEG CH_2_CONHC*H*_*2*_), 3.43 (2H, t, *J* = 6.3 Hz, C*H*_*2*_CH_2_CH_2_NH_3_), 3.49–3.60 (10H, m, OC*H*_*2*_C*H*_*2*_O (8H), NHCH_2_CH_2_C*H*_*2*_), 3.80 (2H, t, *J* = 7.1 Hz, SO_2_C*H*_*2*_), 3.82–3.88 (1H, m, C*H* cyclooctane), 6.65 (1H, br s, N*H* cyclooctylamine), 8.15 (1H, t, *J* = 5.7, CH_2_CON*H*), 8.39 (3H, s, N*H*_*3*_), 8.42 (2H, s, N*H*_*2*_SO_2_).

^19^F NMR (376 MHz, DMSO-*d*_6_): δ −125.00 (C3–*F*, s), −133.93 (C5–*F*, dd, ^1^*J* = 27.1 Hz, ^2^*J* = 12.4 Hz), −150.58 (C6–*F*, dd, ^1^*J* = 26.9 Hz, ^2^*J* = 6.7 Hz).

HRMS (*m*/*z*): [M]^+^ calcd for C_27_H_46_F_3_N_4_O_8_S_2_^+^, 675.2704; found, 675.2703.

### 4.8 *N*-(35-Amino-3,6,9,12,15,18,21,24,27,30,33-undecaoxapentatriacontyl)-3-((2-(cyclooctylamino)-3,5,6-trifluoro-4-sulfamoylphenyl)sulfonyl)propanamide (**GZ22-3**)

TLC (acetone/EtOAc/MeOH/CH_3_COOH 33:33:33:1 v/v/v/v): *R*_f_ = 0.5. Yield: 118.1 mg, 94%; yellow oily residue;

HRMS (*m*/*z*): [M]^+^ calcd for C_41_H_74_F_3_N_4_O_16_S_2_^+^, 999.4488; found, 999.4483.

### 4.9 General Procedure for the Syntheses of **GZ21-18** and **GZ21-20**

A solution of 2-(3-hydroxyphenyl)acetic acid (87.5 mg, 0.58 mmol, 10 equiv) or 3-(4-hydroxyphenyl)propanoic acid (77.0 mg, 0.47 mmol, 10 equiv for **GZ21-20**) was mixed with 2 mL of water, and 0.41 mL of 1 M Na_2_CO_3_ was added dropwise into the mixture until pH 10. Then, NIR-783 (50.0 mg, 0.07 mmol, 1 equiv or 35.0 mg, 0.05 mmol, 1 equiv for **GZ21-20**) was added, and the mixture was stirred at 70 °C for 24 h. The reaction progress was monitored using TLC. The solvent was evaporated under reduced pressure, and the residue was purified with column chromatography.

### 4.10 Sodium 4-((*Z*)-2-((*E*)-2-(2-(3-(Carboxymethyl)phenoxy)-3-((*E*)-2-(3,3-dimethyl-1-(4-sulfonatobutyl)-3*H*-indol-1-ium-2-yl)vinyl)cyclohex-2-en-1-ylidene)ethylidene)-3,3-dimethylindolin-1-yl)butane-1-sulfonate (**GZ21-18**)

TLC (MeOH/acetone/CHCl_3_, 1:1:1 v/v/v): *R*_f_ = 0.5. Purified with column chromatography (gradient: MeOH/acetone/EtOAc, 1:1:1 v/v/v → MeOH). Yield: 62.0 mg, 78%; mp 226–228 °C;

^1^H NMR (400 MHz, DMSO-*d*_6_): δ 1.29 (12H, s, C*H*_*3*_), 1.65–1.81 (8H, m, NCH_2_C*H*_*2*_C*H*_*2*_), 1.89–1.97 (2H, m, OCCCH_2_C*H*_*2*_), 2.44–2.54 (4H, m (inside DMSO peak), OCCC*H*_*2*_), 2.72 (4H, t, *J* = 6.0 Hz, SO_3_C*H*_*2*_), 3.33 (2H, s, C*H*_*2*_COOH), 4.12 (4H, t, *J* = 7.2 Hz, NC*H*_*2*_), 6.21 (2H, d, *J* = 14.2 Hz, OCCC*H*), 6.84 (1H, dd, *J* = 8.0, 2.6 Hz, OCC*H*CH), 6.96 (1H, d, *J* = 7.5 Hz, OCCHCHC*H*), 7.13 (1H, s, OCC*H*C), 7.15–7.25 (3H, m, indolene), 7.31–7.42 (4H, m, indolene), 7.50 (2H, d, *J* = 7.4 Hz, OCCHC*H*CH), 7.86 (2H, d, *J* = 14.1 Hz, OCCCHC*H*), acid peak inside H_2_O.

^13^C NMR (100 MHz, DMSO-*d*_6_): δ 22.99, 23,94, 24.25, 26.47, 27.71, 40.84, 43.98, 45.57, 49.01, 51.17, 100.63, 111.55, 111.68, 116.07, 122.20, 122.80, 123.96, 125.07, 128.88, 129.79, 141.54, 142.55, 159.67, 163.61, 171.93, 174.20, 174.42.

HRMS (*m*/*z*): [M]^−^ calcd for C_46_H_53_N_9_O_10_S_2_^–^, 841.3197; found, 841.3114.

### 4.11 Sodium 4-((*Z*)-2-((*E*)-2-(2-(4-(2-Carboxyethyl)phenoxy)-3-((*E*)-2-(3,3-dimethyl-1-(4-sulfonatobutyl)-3*H*-indol-1-ium-2-yl)vinyl)cyclohex-2-en-1-ylidene)ethylidene)-3,3-dimethylindolin-1-yl)butane-1-sulfonate (**GZ21-20**)

TLC (MeOH/acetone/EtOAc, 1:1:1 v/v/v): *R*_f_ = 0.1. Purified with column chromatography (gradient: MeOH/acetone/EtOAc, 1:1:1 v/v/v → MeOH). Yield: 37.0 mg, 90%; mp 225–228 °C;

^1^H NMR (400 MHz, DMSO-*d*_6_): δ 1.25 (12H, s, *CH*_*3*_), 1.62–1.79 (8H, m, NCH_2_C*H*_*2*_C*H*_*2*_), 1.88–1.97 (2H, m, OCCCH_2_C*H*_*2*_), 2.10 (2H, t, *J* = 7.8 Hz, C*H*_*2*_CH_2_COOH), 2.36–2.56 (4H, m (inside DMSO peak), OCCC*H*_*2*_), 2.63–2.77 (6H, m, C*H*_*2*_COOH and SO_3_C*H*_*2*_), 4.12 (4H, t, *J* = 7.8 Hz, NC*H*_*2*_), 6.21 (2H, d, *J* = 14.2 Hz, OCCC*H*), 7.02 (2H, d, *J* = 8.5 Hz, OCC*H*), 7.14–7.24 (4H, m, indolene), 7.31–7.41 (4H, m, indolene), 7.49 (2H, d, *J* = 7.4 Hz, OCCHC*H*), 7.82 (2H, d, *J* = 14.1 Hz, OCCCHC*H*), acid peak inside H_2_O.

^13^C NMR (100 MHz, DMSO-*d*_6_): δ 22.99, 24.18, 24.98, 26.48, 27.67, 31.94, 43.96, 48.96, 49.04, 51.17, 100.65, 111.66, 114.56, 122.04, 122.82, 125.12, 126.73, 128.94, 130.37, 137.42, 141.41, 142.54, 158.15, 163.22, 171.86, 175.83.

HRMS (*m*/*z*): [M]^−^ calcd for C_47_H_55_N_2_O_9_S_2_^–^, 855.3354; found, 855.3327.

### 4.12 Sodium 4-((*Z*)-2-((*E*)-2-(2-(3-(20-((2-(Cyclooctylamino)-3,5,6-trifluoro-4-sulfamoylphenyl)sulfonyl)-2,18-dioxo-7,10,13-trioxa-3,17-diazaicosyl)phenoxy)-3-((*E*)-2-(3,3-dimethyl-1-(4-sulfonatobutyl)-3*H*-indol-1-ium-2-yl)vinyl)cyclohex-2-en-1-ylidene)ethylidene)-3,3-dimethylindolin-1-yl)butane-1-sulfonate (**GZ21-19**)

A mixture of **GZ21-18** (18.0 mg, 20.8 μmol, 1 equiv), **GZ21-10** (15.0 mg, 22.8 μmol, 1.1 equiv), EDC (10.7 mg, 62.4 μmol, 3 equiv), HOBT (6.0 mg, 41.6 μmol, 2 equiv), 0.5 mL of dry pyridine, and 3 mL of DMSO was stirred at room temperature under an argon atmosphere for 48 h. The reaction progress was monitored using TLC (EtOAc/acetone/MeOH, 2:2:3 v/v/v): *R*_f_ = 0.7. The solvent was evaporated under reduced pressure, then the crude product was poured into saturated sodium chloride solution (10 mL), acidified until neutral pH, and extracted with ethyl acetate. The organic layer was washed with sat. sodium chloride solution and dried over sodium sulfate. The solvent was evaporated under reduced pressure, and the residue was purified with column chromatography (MeOH/EtOAc/acetone, 1:1:1 v/v/v). Yield: 2.0 mg, 6%;

^1^H NMR (400 MHz, DMSO-*d*_6_): δ 1.25 (12H, s, *CH*_*3*_), 1.38–1.91 (28H, m, (4H NCH_2_C*H*_*2*_CH_2_, 2H OCCCH_2_C*H*_*2*_, 8H SO_3_CH_2_C*H*_*2*_C*H*_*2*_ and 14H cyclooctane)), 2.37–2.72 (6H, m, SCH_2_C*H*_*2*_CONH and OCCC*H*_*2*_ (overlapping DMSO residual peak)), 2.66–2.76 (4H, m, SO_3_C*H*_*2*_), 2.92–3.11 (6H, m, PEG C*H*_*2*_NH and CC*H*_*2*_CON), 3.20–3.97 (15H, m, SO_2_C*H*_*2*_, C*H* cyclooctane and PEG), 4.12 (4H, s, NC*H*_*2*_), 6.22 (2H, d, *J* = 14.2 Hz, OCCC*H*), 6.55–6.65 (1H, m, N*H* cyclooctylamine), 6.89–7.55 (12H, m, phenol, indolene), 7.81 (2H, d, *J* = 14.6 Hz, OCCCHC*H*), 8.04–8.24 (2H, m, N*H*CO).

^19^F NMR (376 MHz, DMSO-*d*_6_): δ −125.00 (C3–*F*, s), −133.97 (C5–*F*, d, *J* = 27.1 Hz), −150.58 (C6–*F*, d, *J* = 24.1 Hz).

HRMS (*m*/*z*): [M]^+^ calcd for C_73_H_98_F_3_N_4_O_16_S_4_^+^, 1499.5869; found, 1499.5870.

### 4.13 General Procedure for the Syntheses of **GZ22-1** and **GZ22-4**

A mixture of **GZ21-20** (20.0 mg, 22.7 μmol, 1 equiv), **GZ21-10** (16.9 mg, 25.0 μmol, 1.1 equiv) or **GZ22–3** (25.0 mg, 25.0 μmol, 1.1 equiv) for **GZ22-4**, EDC (8.8 mg, 56.8 μmol, 2.5 equiv), HOBT (6.1 mg, 45.5 μmol, 2 equiv), 0.5 mL of dry pyridine, and 3 mL of DMF was stirred at room temperature under an argon atmosphere for 24 h. The reaction progress was monitored using TLC. The solvent was evaporated under reduced pressure, and the crude product was poured into saturated sodium chloride solution (10 mL), acidified until neutral pH, and extracted with ethyl acetate. The organic layer was washed with sat. sodium chloride solution and dried over sodium sulfate. The solvent was evaporated under reduced pressure and residue was purified with column chromatography.

### 4.14 Sodium 4-((*Z*)-2-((*E*)-2-(2-(4-(21-((2-(Cyclooctylamino)-3,5,6-trifluoro-4-sulfamoylphenyl)sulfonyl)-3,19-dioxo-8,11,14-trioxa-4,18-diazahenicosyl)phenoxy)-3-((*E*)-2-(3,3-dimethyl-1-(4-sulfonatobutyl)-3*H*-indol-1-ium-2-yl)vinyl)cyclohex-2-en-1-ylidene)ethylidene)-3,3-dimethylindolin-1-yl)butane-1-sulfonate (**GZ22-1**)

TLC (acetone/MeOH/EtOAc, 1:1:1 v/v/v): *R*_f_ = 0.4. Purified with column chromatography (acetone/MeOH/EtOAc, 1:1:1 v/v/v). Yield: 5.1 mg, 15%;

^1^H NMR (400 MHz, DMSO-*d*_6_): δ 1.31 (12H, s, *CH*_*3*_), 1.44–0.04 (28H, m, NCH_2_C*H*_*2*_CH_2_ (4H), OCCCH_2_C*H*_*2*_, SO_3_CH_2_C*H*_*2*_C*H*_*2*_ (8H) and cyclooctane (14H)), 2.27–2.35 (2H, m, CC*H*_*2*_CH_2_CO), 2.49–2.60 (4H, m, OCCC*H*_*2*_ (overlapping DMSO residual peak)), 2.65 (2H, t, *J* = 6.8 Hz, SCH_2_C*H*_*2*_CONH), 2.71–2.82 (4H, m, SO_3_C*H*_*2*_), 3.01–3.11 (4H, m, PEG C*H*_*2*_NH), 3.32–4.12 (17H, m, SO_2_C*H*_*2*_, CC*H*_*2*_CON, C*H* cyclooctane and PEG), 4.14–4.22 (4H, s, NC*H*_*2*_), 6.27 (2H, d, *J* = 14.2 Hz, OCCC*H*), 6.64 (1H, d, *J* = 8.8 Hz, N*H* cyclooctylamine), 7.06–7.13 (2H, m, phenol), 7.20–7.33 (4H, m, indolene), 7.36–7.46 (4H, m, indolene), 7.54 (2H, d, *J* = 7.5 Hz, phenol), 7.87 (2H, d, *J* = 14.0 Hz, OCCCHC*H*), 7.98 (1H, t, *J* = 5.7 Hz, SCH_2_CH_2_CON*H*), 8.20–8.31 (1H, m, N*H*COCH_2_CH_2_C).

^19^F NMR (376 MHz, DMSO-*d*_6_): δ −125.00 (C3–*F*, s), −133.97 (C5–*F*, d, *J* = 27.1 Hz), −150.58 (C6–*F*, d, *J* = 24.1 Hz).

HRMS (*m*/*z*): [M]^−^ calcd for C_74_H_98_F_3_N_6_O_16_S_4_^–^, 1511.5879; found, 1511.5818.

### 4.15 Sodium 4-((*Z*)-2-((*E*)-2-(2-(4-(43-((2-(Cyclooctylamino)-3,5,6-trifluoro-4-sulfamoylphenyl)sulfonyl)-3,41-dioxo-7,10,13,16,19,22,25,28,31,34,37-undecaoxa-4,40-diazatritetracontyl)phenoxy)-3-((*E*)-2-(3,3-dimethyl-1-(4-sulfonatobutyl)-3*H*-indol-1-ium-2-yl)vinyl)cyclohex-2-en-1-ylidene)ethylidene)-3,3-dimethylindolin-1-yl)butane-1-sulfonate (**GZ22-4**)

TLC (acetone/MeOH/AcOH, 33:66:1 v/v/v): *R*_f_ = 0.3. Purified with column chromatography (MeOH/acetone, 1:3 v/v). Yield: 4.0 mg, 9%

^1^H NMR (400 MHz, DMSO-*d*_6_): δ 1.26 (12H, s, *CH*_*3*_), 1.39–1.88 (22H, m, (8H NCH_2_C*H*_*2*_C*H*_*2*_ and 14H cyclooctane)), 1.89–1.98 (2H, m, OCCCH_2_C*H*_*2*_), 2.27 (2H, t, *J* = 8.0 Hz, CC*H*_*2*_CH_2_CON), 2.42–2.54 (4H, m (overlapping DMSO residual peak), OCCC*H*_*2*_), 2.60 (2H, t, *J* = 7.1 Hz, SCH_2_C*H*_*2*_CONH), 2.65–2.77 (6H, m, CCH_2_C*H*_*2*_CON and SO_3_C*H*_*2*_), 3.08–3.17 (4H, m, PEG C*H*_*2*_NH), 3.49 (44H, s, PEG), 3.73 (2H, t, *J* = 7.1 Hz, SO_2_C*H*_*2*_), 3.75–3.82 (1H, m, C*H* cyclooctane), 4.13 (4H, t, *J* = 7.2 Hz, NC*H*_*2*_), 6.22 (2H, d, *J* = 14.2 Hz, OCCC*H*), 6.59 (1H, d, *J* = 7.9 Hz, N*H* cyclooctylamine), 7.06 (2H, d, *J* = 8.1 Hz, OCC*H*), 7.15–7.24 (4H, m, indolene), 7.32–7.42 (4H, m, indolene), 7.48 (2H, d, *J* = 7.5 Hz, OCCHC*H*), 7.82 (2H, d, *J* = 13.9 Hz, OCCCHC*H*), 7.83–7.88 (1H, m, N*H*COCH_2_CH_2_C), 8.15 (1H, t, *J* = 5.3, SO_2_CH_2_CH_2_CON*H*).

^19^F NMR (376 MHz, DMSO-*d*_6_): δ −125.02 (C3–*F*, s), −134.05 (C5–*F*, dd, ^1^*J* = 27.2 Hz, ^2^*J* = 12.5 Hz), −150.56 (C6–*F*, dd, ^1^*J* = 27.1 Hz, ^2^*J* = 6.4 Hz).

HRMS (*m*/*z*): [M]^+^ calcd for C_88_H_128_F_3_N_6_O_24_S_4_^+^, 1837.7810; found, 1837.7844.

### 4.16 *tert*-Butyl (1-((2-(Cyclooctylamino)-3,5,6-trifluoro-4-sulfamoylphenyl)sulfonyl)-4,7-dioxo-12,15,18-trioxa-3,8-diazahenicosan-21-yl)carbamate (**AZ21-4**)

Commercially available 2,2-dimethyl-4,20-dioxo-3,9,12,15-tetraoxa-5,19-diazatricosan-23-oic acid (93 mg, 0.22 mmol) was dissolved in DMF (2 mL) and pyridine (1 mL), then EDC (58 mg, 0.30 mmol) was added, and the reaction mixture was stirred for 1 h at room temperature, under an argon atmosphere. Then, 4-((2-aminoethyl)sulfonyl)-3-(cyclooctylamino)-2,5,6-trifluorobenzenesulfonamide hydrochloride (AZ21-1B) (114 mg, 0.24 mmol) was added, and the reaction mixture was stirred for 24 h at room temperature, under an argon atmosphere. The solvents were removed at reduced pressure, and the resultant precipitate was dissolved in ethyl acetate. The organic extract was washed with 5% HCl(aq), then with 5% NaHCO_3_, and dried over sodium sulfate. The solvent was evaporated under reduced pressure, and the residue was purified with column chromatography (ethyl acetate/MeOH (5:1), *R*_f_ = 0.7). Yield: 116.0 mg, 62%, yellow oily residue.

^1^H NMR (400 MHz, DMSO-*d*_6_): δ 1.37 (9H, s, C(C*H*_*3*_)_3_), 1.42–1.68 (16H, m, cyclooctane (12H) and OCH_2_C*H*_*2*_CH_2_NH (4H)), 1.81–1.88 (2H, m, cyclooctane), 2.18–2.27 (4H, HNCOC*H*_*2*_C*H*_*2*_CONH), 2.96 (2H, q, *J* = 6.4 Hz, CONHC*H*_*2*_CH_2_CH_2_O), 3.06 (2H, q, *J* = 6.4 Hz, CONHC*H*_*2*_CH_2_CH_2_O), 3.38 (4H, t, *J* = 6.0 Hz, NHCH_2_CH_2_C*H*_*2*_O), 3.41–3.53 (10H, m, OC*H*_*2*_C*H*_*2*_O (8H) and CONHC*H*_*2*_CH_2_SO_2_), 3.68 (2H, t, *J* = 6.4 Hz, SO_2_C*H*_*2*_), 3.77 (1H, br s, C*H* cyclooctane), 6.60 (1H, d, *J* = 8.0 Hz, N*H*-cyclooctyl), 6.76 (1H, t, *J* = 5.6 Hz, N*H*COO), 7.79 (1H, t, *J* = 5.6 Hz, N*H*CO), 8.06 (1H, t, *J* = 5.2 Hz, CON*H*CH_2_CH_2_SO_2_), 8.36 (2H, s, SO_2_N*H*_*2*_).

^19^F NMR (376 MHz, DMSO-*d*_6_): δ −124.7 (C2–*F*, s), −134.4 (C6–*F*, dd, ^1^*J* = 26.9 Hz, ^2^*J* = 12.9 Hz), −150.2 (C5–*F*, dd, ^1^*J* = 27.0 Hz, ^2^*J* = 6.4 Hz).

HRMS for C_35_H_58_F_3_N_5_O_11_S_2_ [(M + H)^+^]: calcd, 846.3599; found, 846.3613.

### 4.17 Sodium 4-((*Z*)-2-((*E*)-2-(2-((1-((2-(Cyclooctylamino)-3,5,6-trifluoro-4-sulfamoylphenyl)sulfonyl)-4,7-dioxo-12,15,18-trioxa-3,8-diazahenicosan-21-yl)amino)-3-((*E*)-2-(3,3-dimethyl-1-(4-sulfonatobutyl)-3*H*-indol-1-ium-2-yl)vinyl)cyclohex-2-en-1-ylidene)ethylidene)-3,3-dimethylindolin-1-yl)butane-1-sulfonate (**AZ21-6**)

**AZ21-4** (32 mg, 0.038 mmol) was dissolved in dichloromethane (7 mL) and TFA (0.3 mL), and the reaction mixture was stirred for 2 h at room temperature. The solvents were removed at reduced pressure, and the resultant precipitate was dissolved in DMF (3 mL). Then, NIR-783 (33 mg, 0.044 mmol) and DIPEA (50 μL, 0.287 mmol) were added, and the reaction mixture was stirred for 24 h at room temperature under an argon atmosphere. The solvents were removed at reduced pressure, and the residue was purified with column chromatography (chloroform/ethyl acetate/MeOH (1:1:1), *R*_f_ = 0.37). Yield: 27 mg, 49%.

^1^H NMR (400 MHz, DMSO-*d*_6_): δ 1.42–1.77 (34H, m, C(C*H*_*3*_)_2_ (12H), cyclooctane (12H), C*H*_*2*_(IR-dye) (6H) and OCH_2_C*H*_*2*_CH_2_NH (4H)), 1.79–1.88 (6H, m, cyclooctane (2H) and C*H*_*2*_(IR-dye) (4H), 2.16–2.26 (4H, HNCOC*H*_*2*_C*H*_*2*_CONH), 2.48 (4H, m, C*H*_*2*_(IR-dye) overlapping with DMSO), 3.02 (4H, m, NHC*H*_*2*_CH_2_CH_2_O), 3.30–3.55 (14H, m, NHCH_2_CH_2_C*H*_*2*_O (4H), (OC*H*_*2*_C*H*_*2*_O)_2_ and CONHC*H*_*2*_CH_2_SO_2,_ overlapping with H_2_O), 3.63–3.70 (2H, m, SO_2_C*H*_*2*_), 3.76 (1H, br s, C*H* cyclooctane), 3.83–3.94 (4H, m, C*H*_*2*_SO_3_), 5.71 (2H, d, *J* = 8.4 Hz, NHCCC*H*), 6.57 (1H, d, *J* = 8.0 Hz, N*H*-cyclooctyl), 7.00 (2H, t, *J* = 7.6 Hz, indolene),7.09 (2H, d, *J* = 8.4 Hz, indolene), 7.25 (2H, t, *J* = 7.6 Hz, indolene), 7.39 (2H, d, *J* = 7.6 Hz, indolene), 7.47 (2H, d, *J* = 9.6 Hz, NHCCCHC*H*), 7.85 (1H, s, N*H*CO), 8.09 (1H, s, CON*H*CH_2_CH_2_SO_2_).

^19^F NMR (376 MHz, DMSO-*d*_6_): δ −124.8 (C2–*F*, s), −134.7 (C6–*F*, d, *J* = 24.1 Hz), −150.4 (C5–*F*, d, *J* = 25.6 Hz).

HRMS for C_68_H_95_F_3_N_7_O_15_S_4_ [(M-H)^−^]: calcd, 1434.5727; found, 1434.5660.

### 4.18 Preparation of Recombinant CA Isozymes

All 12 catalytically active human CA isozymes were prepared recombinantly as previously described. Most isozymes were truncated, and only the catalytic domains of the proteins were produced.^9,56^ All isozymes were expressed in a bacterial expression system except that CAIX was expressed in mammalian cells. The proteins were chromatographically purified via immobilized metal affinity chromatography, ion-exchange, or *p*-aminomethylbenzenesulfonamide-sepharose affinity chromatography. Protein purity was confirmed by SDS-PAGE, and MW was confirmed by mass spectrometry with 1 Da precision, as predicted from theoretical protein sequence.

### 4.19 Fluorescence-Based Thermal Shift Assay

The fluorescence-based thermal shift assay (FTSA) was used to determine the binding affinity of compounds for all catalytically active CA isozymes. The assay is based on the thermal stabilization of proteins by bound ligands and distinguishes itself in its ability to determine extremely high affinity (picomolar *K*_d_) of compound binding to CAIX. The experiments were performed with a QIAGEN Rotor-Gene Q instrument using either the blue channel (365 ± 20 nm excitation and 460 ± 15 nm emission detection) or the green channel (470 ± 10 nm excitation and 510 ± 5 nm detection). The protein solution, in the absence and presence of various compound concentrations ranging from 3 to 200 μM (2× dilutions), was heated from 25 to 99 °C (heating rate, 1 °C/min). The CA isozyme melting temperature *T*_m_ was determined by following the fluorescence of 8-anilino-1-naphthalenesulfonate (ANS) or Glomelt dye. The samples consisted of 5 μM protein (or 10 μM CAIV), different concentrations of the tested compound, and 50 μM ANS or 200× diluted Glomelt in 50 mM sodium phosphate buffer (at pH 7.0) containing 100 mM sodium chloride and 2–4% (v/v) dimethyl sulfoxide. To obtain the dissociation constant (at 37 °C), data analysis was performed, and the curves were fit using the Thermott Web server.^57^

### 4.20 Construction of the CAIX Expression Plasmid

The cDNA of human carbonic anhydrase IX (CAIX) in pOTB7 vector was purchased from RZPD Deutsches Ressourcenzentrum für Genomforschung GmbH (Germany). For the construction of the mammalian CAIX expression plasmid pCMV-CAIX, the cDNA fragment, encoding full-length CAIX sequence, was cut out from the pOTB7-CAIX plasmid using restriction endonucleases PsiI and Ppu21I (partial digestion) and inserted into the pCMV-HA vector (Clontech) via digested ApaI and XhoI restriction sites, blunted by Klenow fragment. The pCMV-CAIX expression plasmid contains an open reading frame for the full-length CAIX protein (1–459 amino acids) without a tag because the HA tag, originally present in the pCMV-HA vector, was removed during the subcloning procedure.

### 4.21 Measurement of Compound Binding to Live Cells and Compound Competition Assay

Both assays were performed as previously described.^16^ In brief, to measure fluorescent compound binding to CAIX expressed in live cells, HeLa cells were seeded in 12-well plates and either incubated in hypoxia for 3 days or transfected with the CAIX-coding plasmid pCMV-CAIX next day after seeding. The TurboFect transfection reagent (Thermo Fisher) was used for transfection. After 3 days in hypoxia or next day after transfection in normoxia, the cell medium from each well was removed and replaced by 200 μL of a serially diluted NIR-fluorescent compound (12 2-fold dilution steps) in the PBS medium, starting from 4 μM (4, 2, 1,..., 0 nM). Plates were incubated in a CO_2_ incubator at normoxia for 20 min. The solution was removed, and cells were washed 3 times for 1–2 min with 400 μL of PBS. Then, 180 μL of TrypLe express enzyme (Thermo Fisher) was added to each well. After 10 min at normoxia, 20 μL of Defined Trypsin Inhibitor solution (Thermo Fisher) was added and cells were resuspended by pipetting. 150 μL of the suspension from each well was transferred to Thermo Scientific Nunc MicroWell 96-Well Optical-Bottom Plates for fluorescence and absorbance measurements. 759 nm excitation and 800 nm emission wavelengths were used to measure fluorescence on a Synergy HTX, BioTek plate reader. 650 nm wavelength was used for absorbance measurements.

For the competition assay, live cells were seeded in 12-well plates and incubated for 3 days under hypoxia. 200 μL of serially diluted NIR-fluorescent compound (12 2-fold dilution steps) in the PBS medium, starting from 5120 nM (5120, 2560, 1280,..., 0 nM), was mixed with 200 μL of 20 nM **GZ19-32** solution (PBS). After the removal of growth medium, 200 μL obtained solutions were applied to the cells grown in 12-well culture plates. Plates were incubated in the CO_2_ incubator at normoxia for 20 min. The solution was removed, and cells were washed 3 times for 1–2 min with 400 μL of PBS. Then, 180 μL of TrypLe express enzyme (Thermo Fisher) was added to each well and incubated in the normoxia chamber for 10 min. Then, 20 μL of Defined Trypsin Inhibitor solution (Thermo Fisher) was added and cells were resuspended by pipetting. 150 μL of the suspension from each well was transferred to Thermo Scientific Nunc MicroWell 96-Well Optical-Bottom Plates for fluorescence and absorbance measurements. 485 nm excitation and 520 nm emission wavelengths were used to measure fluorescence on a Synergy HTX, BioTek plate reader. 650 nm wavelength was used for absorbance measurements.

### 4.22 Cell Culture and Staining of Live Cells

Human cervical adenocarcinoma cells (HeLa) were kindly provided by Dr. A. Kanopka (Vilnius University). The CAIX-knockout cell line (HeLaCAIX^KO^) was developed using the CRISPR-Cas9 knockout system as described previously.^16^ HeLa cells were cultured in Dulbecco’s modified Eagle’s medium (DMEM) with GlutaMAX (Gibco, Thermo Fisher), supplemented with 10% of heat-inactivated fetal bovine serum, 100 units/mL penicillin, and 100 mg/mL streptomycin in a humidified atmosphere at 37 °C and 5% CO_2_. Hypoxia, when needed, was achieved in the humidified CO_2_ incubator with oxygen control (Binder, Germany), using conditions of 1% O_2_, 5% CO_2_, and 94% N_2_ and 37 °C. Cells were regularly checked for mycoplasma contamination using a SouthernBiotech Mycoplasma Detection Kit (#OB1310001) according to manufacturer’s directions.

For visualization of CAIX in live cells, HeLa cells were seeded in 12-well plates, and the next day they were transfected with the pCMV-CAIX plasmid to induce CAIX expression under normoxia. The TurboFect Transfection Reagent (Thermo Fisher) was used for this purpose according to manufacturer’s directions. Cells were stained and visualized next day after transfection. Nontransfected cells, grown under normoxia, were used as a control. For the induction of CAIX expression without transfection, cells were cultured under hypoxia for 3 days.

For cell imaging, live cells were incubated with the recombinant monoclonal antibody to CAIX (M75) (Absolute Antibody, #Ab00414–1.1) in the CO_2_ incubator for 30 min. The antibody stock solution (1 mg/mL) was used at a dilution of 1:150 in FluoroBright (FB)(Thermo Fisher) medium. After washing (3 × 5 min with DMEM in RT, gentle rock), cells were incubated with secondary Alexa Fluor 488 goat antimouse antibodies (2 mg/mL, Thermo Fisher), at a dilution of 1:200 in FB, in a CO_2_ incubator for 30 min. After washing (3 × 5 min with DMEM in RT and 2 × 1 min with PBS), cells were incubated with NIR-fluorescent inhibitors (250 nm in PBS) in a CO_2_ incubator for 5–8 min. After washing (2 min + 2 × 1 min with PBS), PBS was replaced by FB, and 15 μL of NucBlue Live Cell Stain ReadyProbes reagent (Thermo Fisher) was added per well. After 5 min, cells were observed and photographed using an automated fluorescence microscope EVOS FL Auto (Thermo Fisher).

### 4.23 Mice

Female nude mice (CR ATH HO Code 24106216), 5–6 weeks old, were obtained from Charles River Laboratories. Animals were housed, bred, and handled in the Department of Animal Models Animal Facility at the Life Sciences Center, Vilnius University, Lithuania, with a 12 h light–dark cycle, at 21–23 °C and 40–60% humidity. Animals were fed with an irradiated cholorophyll-deficient diet (Altromin, #C1086194) and water ad libitum. All experimental procedures conformed to Directive 2010/63/EU requirements and were approved by the Lithuanian State Food and Veterinary Service (Approval no G2-194, 2021-11-09).

Before injection into animals, mycoplasma-free cells in the exponential growth phase were harvested, washed, and resuspended in PBS. 8–12 weeks old female nude mice were inoculated S.Q. with 3 × 10^6^ HeLaCAIX^WT^ cells in 100 μL of PBS into the right flank of the mice, and the same number of HeLaCAIX^KO^ cells were injected into the left flank of the same mouse. Once tumors reached a suitable size for imaging, **GZ22-4** or **AZ21-6** compounds were injected I.V. at a dose of 2 mg/kg/dose once. Mice were imaged every 24 h postinjection using isoflurane anesthesia with an Alliance^TM b^Q9 Imager system. Images were quantified using the UVIBAND MAX analysis software system. At the end of the experiment, animals were euthanized with a flow of 8.0 L/min of medical CO_2_ gas (Elme Messer Lit, Vilnius, Lithuania) followed by cervical dislocation.

## Supporting Information Available

Information on NIR inhibitor absorption and emission spectra, microphotographs of cells stained with **GZ22-1** and **GZ21-19** compounds, comparison at identical exposure conditions, NIR fluorescent inhibitor quantum yields, and ^1^H and ^19^F NMR spectra of **GZ22-4** compound (PDF)

## Abbreviations

CA: carbonic anhydrase
CAIX: carbonic anhydrase isozyme IX
DCM: dichloromethane
DIPEA: diisopropylethylamine
DMF: dimethylformamide
DMSO: dimethyl sulfoxide
EDC: 1-ethyl-3-(3-(dimethylamino)propyl)carbodiimide
FTSA: fluorescence-based thermal shift assay
HeLa: human cervical adenocarcinoma cells
HOBt: hydroxybenzotriazole
IR: infrared
KO: knock out
NMR: nuclear magnetic resonance
PEG: polyethylene glycol
Py: pyridine
TFA: trifluoroacetic acid
TLC: thin-layer chromatography
TMS: tetramethylsilane
TSA: thermal shift assay
WT: wild type
