# Supplementary material for: High-Affinity NIR-Fluorescent Inhibitors for Tumor Imaging via Carbonic Anhydrase IX
Source: Bioconjug Chem. 2024 May 15;35(6):790–803. doi: 10.1021/acs.bioconjchem.4c00144 (PMC11191402; doi:10.1021/acs.bioconjchem.4c00144)
Supplement: Supplementary file 1 — bc4c00144_si_001.pdf [file bc4c00144_si_001.pdf]

Supplementary Materials

High-affinity NIR-fluorescent inhibitors for tumor imaging via carbonic anhydrase IX

Gediminas Žvinys [a], Agne Petrosiute [a], Audrius Zakšauskas [a], Asta Zubrienė [a], Alvilė Ščerbavičienė [b], Zane Kalnina [c], Edita Čapkauskaitė [a], Vaida Juozapaitienė [a], Aurelija Mickevičiūtė [a], Kirill Shubin [d], Švitrigailė Grincevičienė [a], Steponas Raišys [e], Kaspars Tars [c], Jurgita Matulienė [a], and Daumantas Matulis [a*]

[a] Department of Biothermodynamics and Drug Design, Institute of Biotechnology, Life Sciences Center, Vilnius University, Saulėtekio 7, Vilnius, LT-10257, Lithuania

[b] Department of Biological Models, Institute of Biochemistry, Life Sciences Center, Vilnius University, Saulėtekio 7, Vilnius, LT-10257, Lithuania

[c] Latvian Biomedical Research and Study Centre, Ratsupites 1 k-1, Riga, LV-1067, Latvia

[d] Latvian Institute of Organic Synthesis, Aizkraukles 21, Riga, LV-1006, Latvia

[e] Institute of Photonics and Nanotechnology, National center for physical sciences and technology, Vilnius University, Saulėtekio 3, Vilnius, LT-10257, Lithuania

* Corresponding author: Daumantas Matulis

e-mails: daumantas.matulis@bti.vu.lt, matulis@ibt.lt

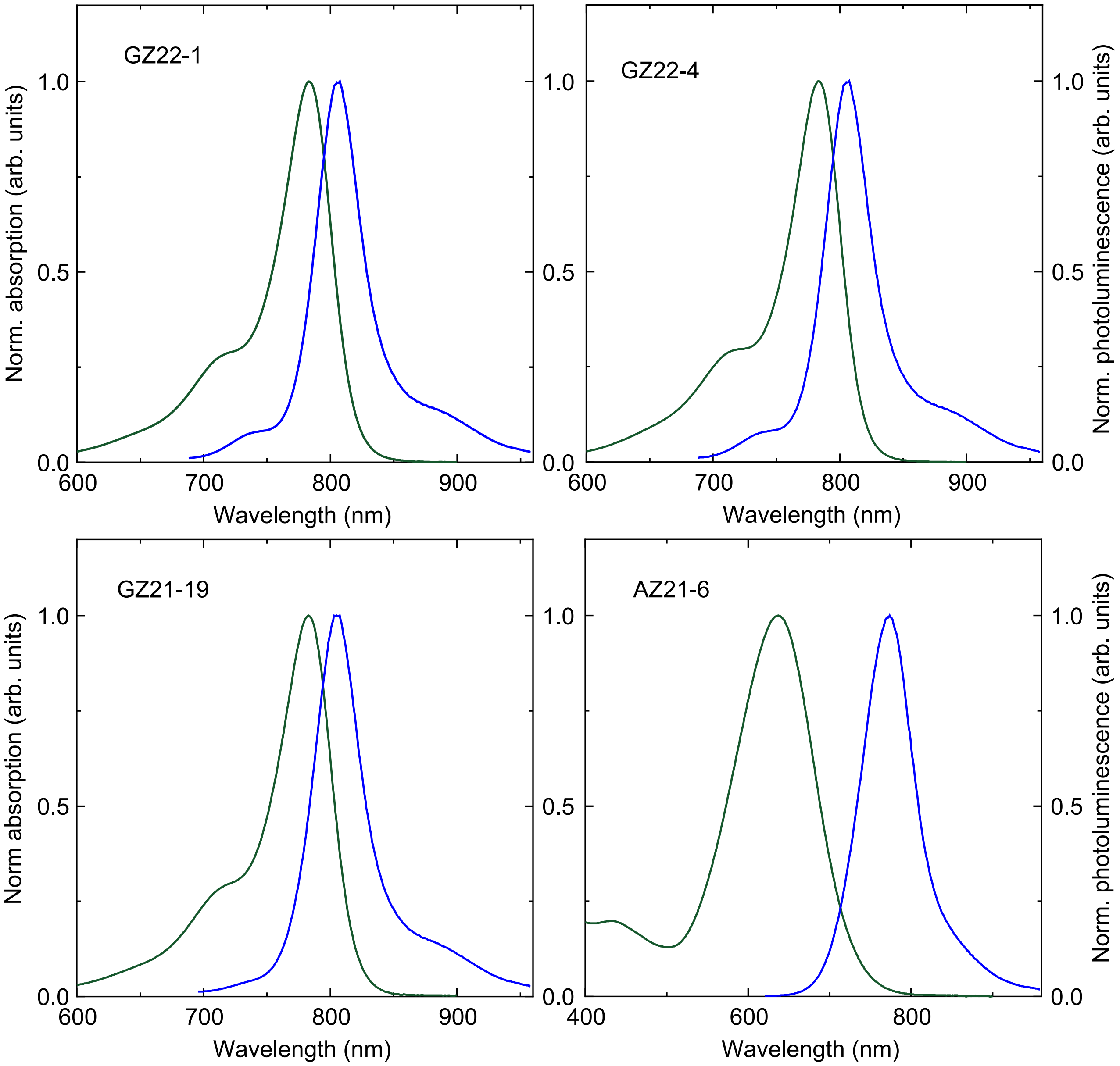

Figure S1. Absorption and emission spectra of NIR inhibitors. GZ22-1 λ_{abs}= 783 nm, λ_{em}= 807 nm; GZ22-4 λ_{abs}= 783 nm, λ_{em}= 807 nm; GZ21-19 λ_{abs}= 783 nm, λ_{em}= 805 nm; AZ21-6 λ_{abs}= 637 nm, λ_{em}= 773 nm.

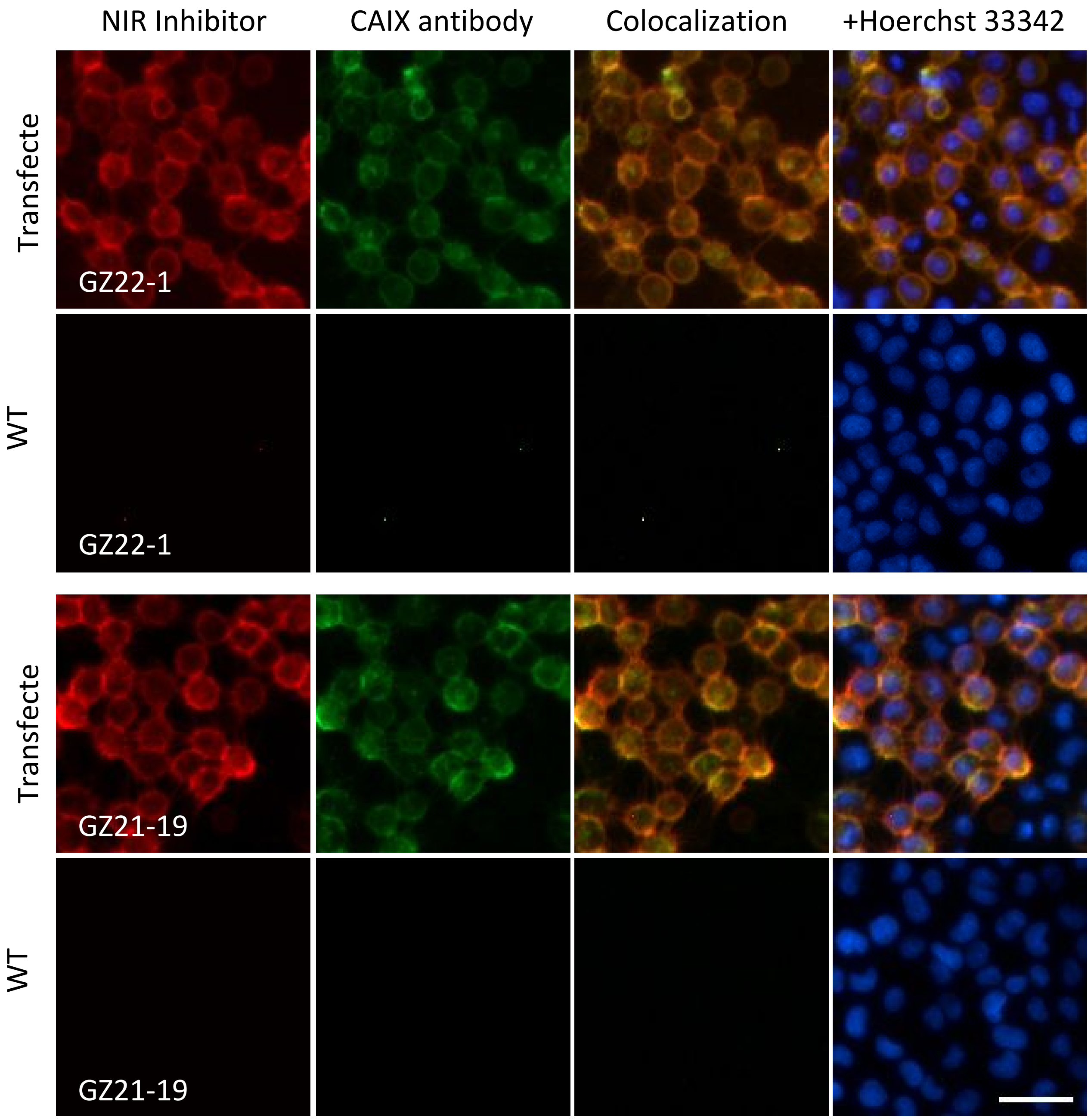

Figure S2. Staining of live HeLa cells grown under normoxia (wild type and transfected with DNA) incubated with 250 nM NIR inhibitors (GZ22-1, GZ21-19). Columns from the left show: binding of NIR inhibitors (red), CAIX antibody (green), colocalization of first two columns, and cell nuclei overlayed on the first two columns (Hoerchst 33342, blue). Scale bar length 40 µm.

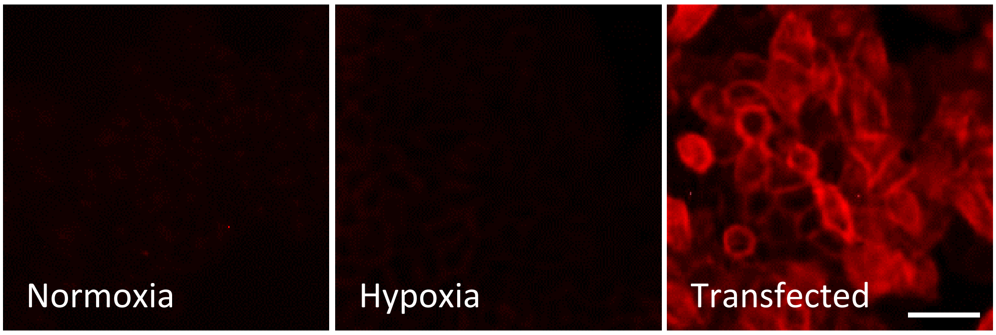

Figure S3. CAIX expressing transfected HeLa cell staining with GZ22-4 NIR-fluorescent compound. Prior to staining the cells were grown for 2 days under normoxia (1st picture), 3 days under hypoxia (2nd picture) and 3rd picture shows cells grown for 1 day under normoxia, then transfected with DNA and incubated for another day under normoxia (2 days total). Compound concentration was 500 nM. All 3 images were taken with Cy7 filter at identical exposure conditions. Scale bar length 40 µm.

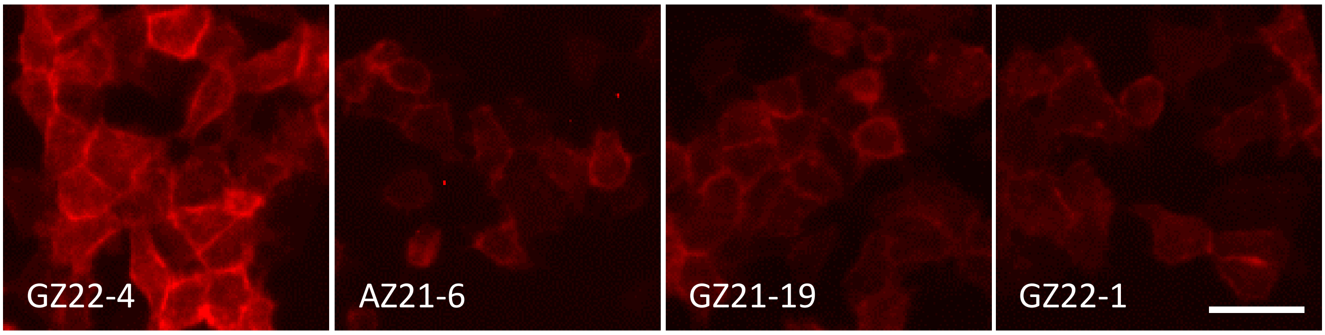

Figure S4. CAIX expressing transfected HeLa cell staining with NIR-fluorescent compounds. Compound concentration was 250 nM. All four images were taken with Cy7 filter at identical exposure conditions. Note that GZ22-4 yielded a brighter staining due to greater fluorescence yield of this inhibitor. Scale bar length 40 µm.

Table S1. Fluorescence quantum yield comparison of NIR-fluorescent compounds. Compound concentration was 100 nM in PBS. $L_{a\text{-}c}$ – excitation radiation integrals, $P_{b\text{-}c}$ – fluorescence integrals.

Nr.	Compound	Excitation	L_a	L_b	L_c	$A^{(1)}$	P_b	P_c	$QY^{(2)}$
1	GZ22-1	780nm	$2.61 \cdot 10^6$	$2.55 \cdot 10^6$	$1.81 \cdot 10^6$	0.29	9756	14369	**0.98**
2	GZ22-4	780nm	$2.60 \cdot 10^6$	$2.48 \cdot 10^6$	$4.12 \cdot 10^5$	0.83	16087	38603	**1.65**
3	GZ21-19	780nm	$2.61 \cdot 10^6$	$2.55 \cdot 10^6$	$1.77 \cdot 10^6$	0.31	12786	31435	**2.82**
4	AZ21-6	650nm	$1.46 \cdot 10^6$	$1.41 \cdot 10^6$	$6.72 \cdot 10^5$	0.52	2987	4731	**0.43**

(1)Sample absorbance $A = 1 - L_c/L_b$. (2)Fluorescence quantum yield: $QY = \frac{P_c - (1-A)P_b}{L_a A}$.

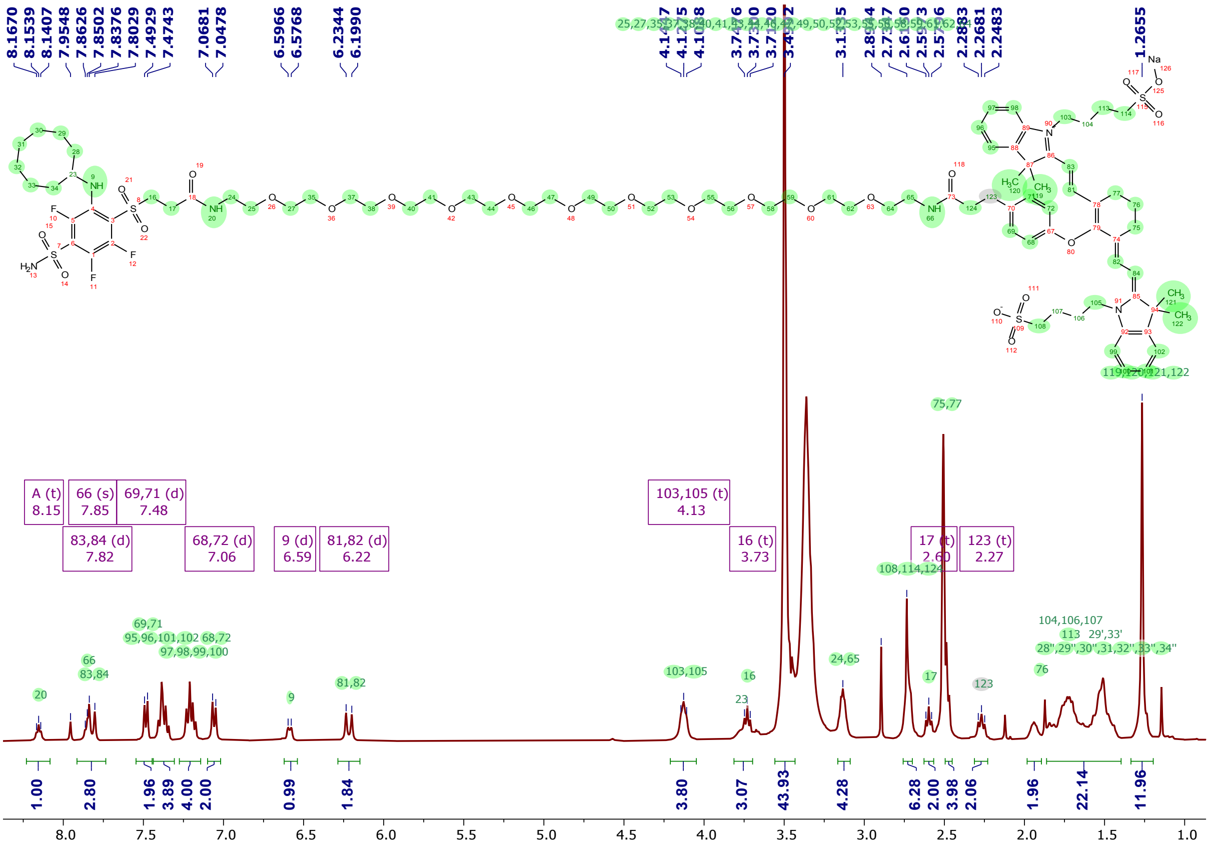

Figure S5. GZ22-4 ^{1}H NMR spectrum. Singlet peaks at 7.95, 2.89 and 2.73 ppm are from dimethylformamide.

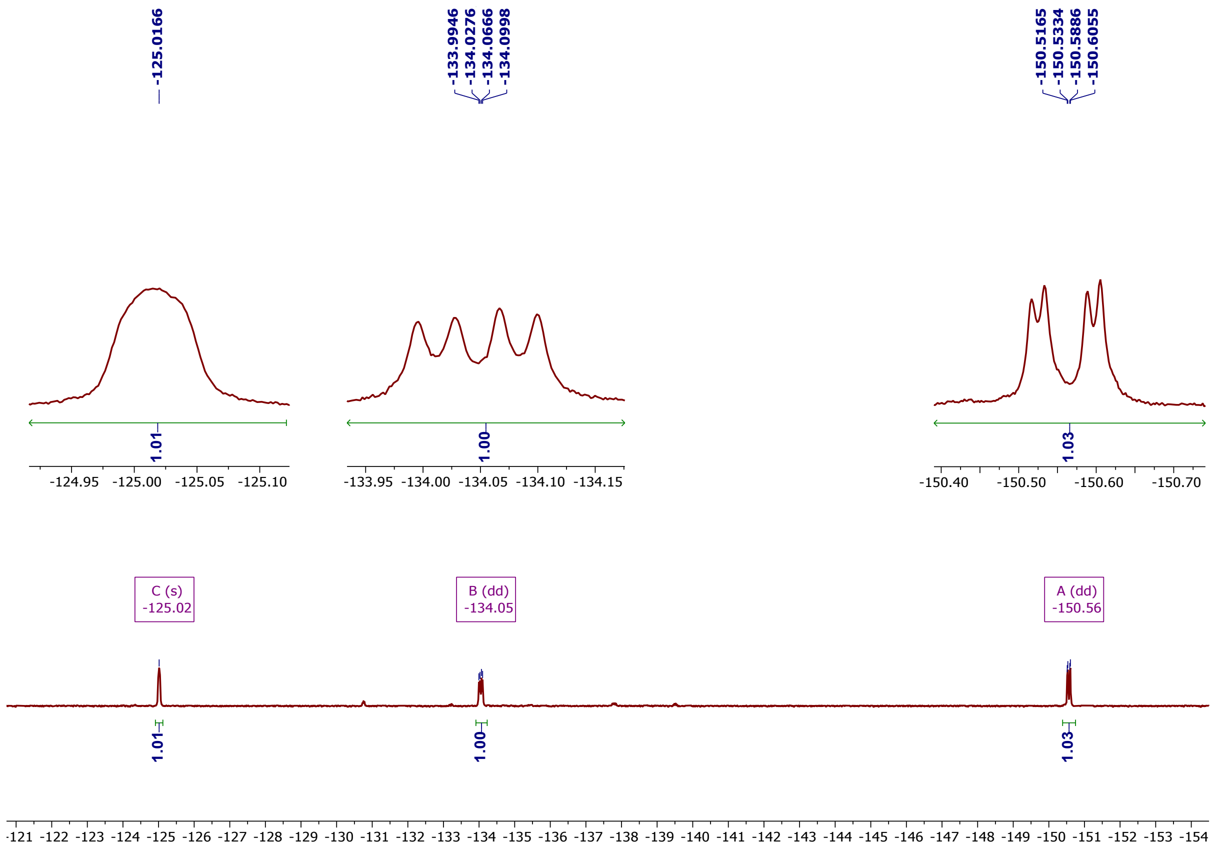

Figure S6. GZ22-4 ^{19}F NMR spectrum.
